# Tandem Probe Analysis Mode for Synchrotron XFM: Doubling Throughput Capacity

**DOI:** 10.1021/acs.analchem.1c04255

**Published:** 2022-03-11

**Authors:** Casey L. Doolette, Daryl L. Howard, Nader Afshar, Cameron M. Kewish, David J. Paterson, Jianyin Huang, Stefan Wagner, Jakob Santner, Walter W. Wenzel, Tom Raimondo, Alexander T. De Vries Van Leeuwen, Lei Hou, Frederik van der Bom, Han Weng, Peter M. Kopittke, Enzo Lombi

**Affiliations:** † Future Industries Institutes, University of South Australia, Mawson Lakes, South Australia 5095, Australia; ‡ Australian Synchrotron, ANSTO, Clayton, Victoria 3168, Australia; § Department of Chemistry and Physics, School of Molecular Sciences, La Trobe University, Melbourne, Victoria 3086, Australia; ∥ UniSA STEM, University of South Australia, Mawson Lakes, South Australia 5095, Australia; ⊥ Chair of General and Analytical Chemistry, Montanuniversität Leoben, Leoben 8700, Austria; # Institute of Analytical Chemistry, University of Natural Resources and Life Sciences Vienna, Tulln 3430, Austria; ¶ Institute of Soil Research, University of Natural Resources and Life Sciences Vienna, Tulln 3430, Austria; ∇ Institute of Agronomy, University of Natural Resources and Life Sciences Vienna, Tulln 3430, Austria; ○ The University of Queensland, School of Agriculture and Food Sciences, St Lucia, Queensland 4072, Australia

## Abstract

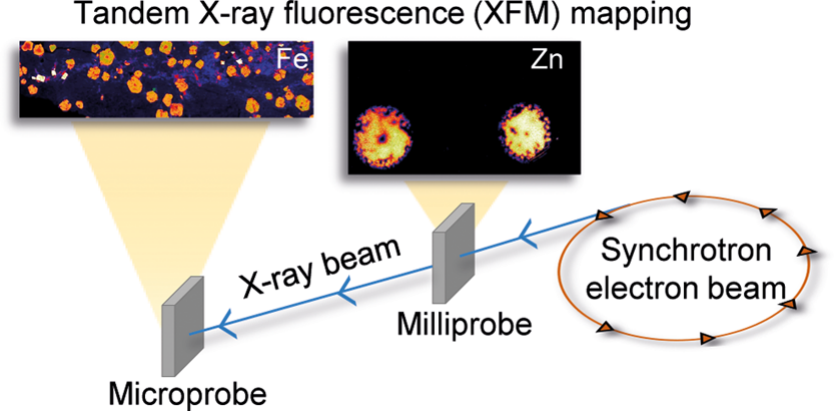

Synchrotron-based X-ray fluorescence microscopy (XFM) analysis is a powerful technique that can be used to visualize elemental distributions across a broad range of sample types. Compared to conventional mapping techniques such as laser ablation inductively coupled plasma mass spectrometry or benchtop XFM, synchrotron-based XFM provides faster and more sensitive analyses. However, access to synchrotron XFM beamlines is highly competitive, and as a result, these beamlines are often oversubscribed. Therefore, XFM experiments that require many large samples to be scanned can penalize beamline throughput. Our study was largely driven by the need to scan large gels (170 cm^2^) using XFM without decreasing beamline throughput. We describe a novel approach for acquiring two sets of XFM data using two fluorescence detectors in tandem; essentially performing two separate experiments simultaneously. We measured the effects of tandem scanning on beam quality by analyzing a range of contrasting samples downstream while simultaneously scanning different gel materials upstream. The upstream gels were thin (<200 μm) diffusive gradients in thin-film (DGT) binding gels. DGTs are passive samplers that are deployed in water, soil, and sediment to measure the concentration and distribution of potentially bioavailable nutrients and contaminants. When deployed on soil, DGTs are typically small (2.5 cm^2^), so we developed large DGTs (170 cm^2^), which can be used to provide extensive maps to visualize the diffusion of fertilizers in soil. Of the DGT gel materials tested (*bis*-acrylamide, polyacrylamide, and polyurethane), polyurethane gels were most suitable for XFM analysis, having favorable handling, drying, and analytical properties. This gel type enabled quantitative (>99%) transmittance with minimal (<3%) flux variation during raster scanning, whereas the other gels had a substantial effect on the beam focus. For the first time, we have (1) used XFM for mapping analytes in large DGTs and (2) developed a tandem probe analysis mode for synchrotron-based XFM, effectively doubling throughput. The novel tandem probe analysis mode described here is of broad applicability across many XFM beamlines as it could be used for future experiments where any uniform, highly transmissive sample could be analyzed upstream in the “background” of downstream samples.

## Introduction

X-ray fluorescence microscopy (XFM, also known as micro-XRF imaging) is a powerful mapping technique that can be used to determine the distribution of elements and chemical species at a range of resolutions. Synchrotron radiation is commonly used as the X-ray source because the photon flux is orders of magnitude greater than that for conventional benchtop XFM, meaning that speed of analysis is also orders of magnitude faster.^1^ There are more than 50 synchrotrons globally, with nearly all having XFM capabilities and some facilities having more than one XFM beamline, such as the National Synchrotron Light Source II and Advanced Photon Source. However, there is high demand for synchrotron-based X-ray fluorescence mapping, and access to synchrotron XFM facilities is often limited by beamtime availability. For instance, the XFM beamline at the Australian Synchrotron (ANSTO) is currently one of the most oversubscribed beamlines at that facility. Therefore, any approach for increasing the throughput of XFM beamlines would be highly valuable to many researchers across several research fields globally. In addition, increasing productivity would also be attractive to synchrotron facilities who can increase outputs of their existing infrastructure investments.

The high demand for synchrotron XFM is driven by two factors: (1) its wide range of applications, including biomedical, geological, environmental, agricultural and cultural heritage fields of research^2^ and (2) the analytical advantages of this technique compared to other methods of visualizing the lateral distribution of elements. Using in situ analysis of plant samples as an example, alternative elemental mapping techniques include autoradiography using radiolabeled elements, laser ablation coupled with inductively coupled plasma mass spectrometry (LA–ICP–MS), confocal microscopy with fluorophores, scanning electron microscopy coupled with energy-dispersive X-ray spectroscopy, and proton-/particle-induced X-ray emission.^3^ The advantages and disadvantages of synchrotron XFM over the aforementioned techniques have been extensively reviewed by Lombi et al.^3^ Briefly, the main advantages of synchrotron XFM are that analyses can be performed at room temperature and pressure with good detection limits (1–100 mg/kg) and with excellent resolution (down to 50 nm).^1,3,4^ Synchrotron XFM can also be used to produce multi-elemental maps (where the energy used to excite the element of interest will also excite the elements with absorption edges of lower energy)^5^ and to scan very large samples (up to ∼1 m^2^).^1^ These two advantages were of particular importance for our study.

The primary goal for our study was to enable, for the first time, the concurrent analysis of two samples on a single XFM beamline. This approach takes advantage of the penetrating nature of X-rays, which allow two samples located in the path of the same beam to be raster scanned simultaneously, and captured and analyzed asynchronously using two detector systems. This outcome was motivated by the need to scan large samples (>100 cm^2^) without sacrificing the overall throughput of the beamline. Large XFM samples can be of biological or mineral origin, artwork or archaeological artefacts, or from molecular biology studies, for example, where synchrotron XFM has been used to characterize the metalloproteins separated on electrophoresis gels.^5−11^ For our study, we focused on gel matrices, but instead of electrophoretic gels, we used diffusive gradients in thin-film (DGT) gels. We chose to investigate gels as they are large objects and, due to their characteristics, unlikely to affect downstream beam quality.

DGT samplers are commonly used to estimate the bioavailable fraction of a nutrient (e.g., phosphate) or contaminant (e.g., lead, cadmium, and cobalt) in environmental systems (i.e., sediment, marine/freshwaters, and soil). The DGT device consists of three layers; a filter membrane that is placed in contact with the sampling surface, a diffusive gel (ion-permeable hydrogel through which the analyte diffuses), and a binding gel (hydrogel containing an analyte-specific binding agent, which immobilizes the analyte).^12,13^ After DGT deployment, the mass of the analyte that accumulates in the binding gel is measured, and a time-averaged flux of the analyte of interest for the deployment time can then be calculated.^14,15^ Although elution of the analyte from the binding layer is the most conventional way to measure its accumulation, quantifiable visualization techniques have also been developed; with a comprehensive review provided by Santner et al.^16^ Such two-dimensional (2D) visualization techniques include LA–ICP–MS,^17−19^ and colorimetric and computer imaging densitometry (CID).^20−23^ These techniques have their limitations. For example, in the context of DGT mapping, the main drawback of LA–ICP–MS is that it is very time-consuming to map large areas (>1 cm^2^) at fine resolution.^16^ Analysis times can be up to several days, making very high-resolution mapping of large areas impractical or in some cases, impossible. In addition, LA–ICP–MS is a destructive technique, meaning that there is a finite number of times the DGT can be analyzed. The CID technique allows for 2D visualization, and quantification, of the analyte in the DGT-binding layer using a conventional flat-bed scanner.^23^ Although this colorimetric technique is fast (relative to LA–ICP–MS), accurate, and low-cost, it has only been developed for few analytes (e.g., sulfide^23,24^ and phosphate^21^), with the main reason being that color-based methods can typically only quantify a single analyte.^16^

To the best of our knowledge, XFM has not been used for mapping analytes in passive sampling devices such as DGTs. Synchrotron-based XFM can overcome the limitations of CID and LA–ICP–MS for DGT mapping, particularly when analyzing large gels. We prepared large DGT gels (170 cm^2^) to visualize the lateral distribution and diffusion of fertilizers in soil; something that is not possible using conventional soil DGTs, which are substantially smaller (2.5 cm^2^). To determine the optimal gel matrix for large DGTs, we evaluated the ease with which six types of DGT could be prepared and handled, and, their suitability for XFM analysis.

The overarching goal of this study was to develop a method to increase the throughput of XFM beamlines at synchrotrons globally. More specifically, the aims of our study were to (a) perform synchrotron XFM elemental mapping of DGTs; (b) evaluate large DGT-binding gels for practical handling properties and suitability for XFM analysis; (c) determine the effect (if any) of various DGT matrices on the X-ray beam downstream of the binding layer; (d) determine the effect of tandem scanning on the microprobe analysis of two highly heterogeneous and contrasting downstream samples; and (e) double the throughput of the XFM beamline by enabling simultaneous scanning of large DGTs and a downstream sample.

## Experimental Section

### Beamline Setup

**Figure 1:**
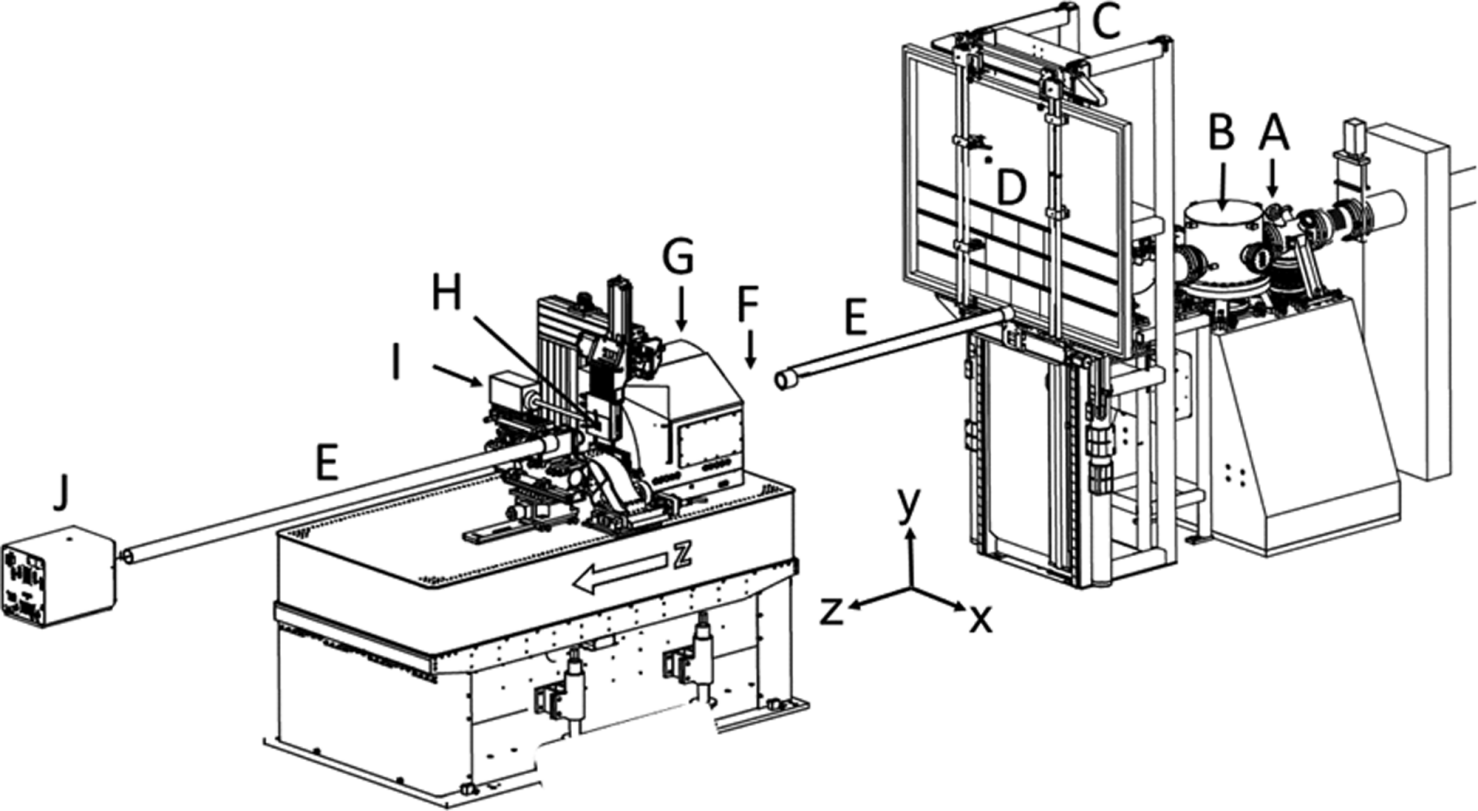
Layout of the XFM beamline end station at the ANSTO. A = quad diode beam position monitor, B = secondary source aperture slits, C = milliprobe, housing upstream Maia detector (hidden by the sample frame), D = sample frame mount with DGT gels, E = removable helium flight tube, F = clean-up slits and ion chamber (not shown for clarity), G = KB focusing mirror enclosure, H = downstream Maia detector sample position, I = silicon drift detector (not used for this experiment), and J = Eiger X-ray detector (not used this experiment). Adapted from Howard et al.^25^ (https://doi.org/10.1107/S1600577520010152) and reproduced with permission of the International Union of Crystallography. A photograph of the beamline is shown in Figure S1.

X-ray fluorescence mapping was performed at the ANSTO at the XFM beamline in Melbourne, Victoria (Figure S1). The optical layout of this beamline, together with a summary of the XFM hardware, is shown in Figure 1. The beamline has two scanning stations, herein referred to as upstream and downstream, both of which were used in this experiment.

### Experimental Design

A summary of the experimental design is reported in Table 1. First, large scans of all DGT-binding layers were collected in the upstream position to determine the best DGT gel sample for mapping the soil distribution of available plant nutrients (Experiment 1). Then, elemental maps of a wheat grain^26^ thin section and a mineral thin section were collected in the downstream position to select the regions of interest for scanning in tandem mode at high resolution (Experiment 2). These two specimens were chosen as representatives of biological and mineral systems; two contrasting sample types commonly analyzed at XFM beamlines. Then, three experiments were performed in tandem mode to assess the effect of scanning various DGTs on downstream analyses of the following: a resolution test pattern (Experiment 3), the mineral sample mentioned above (Experiment 4), and the biological sample mentioned above (Experiment 5).

**Table 1:** Summary of the Experimental Design and the XFM Parameters for Each Experiment

	upstream station					downstream station				
	sample description	size of scanned area (mm)	pixel size (μm × μm)	transit time per pixel (msec)	total time (min)	sample description	size of scanned area (mm)	pixel size (μm x μm)	transit time per pixel (msec)	total time (min)
experiment 1 identification of optimal DGT	DGTs #1 to #6 in two runs	522 × 88	1000 × 1000	20	16	-	-	-	-	-
experiment 2 region of interest search of thin sections	-	-	-	-	-	mineral RB 9B	57 × 13	50 × 50	3.3	20
						wheat grains	43 × 6	20 × 20	2.0	22
experiment 3 effect of the tandem mode on the resolution test pattern	small region of DGTs #1 to #6 (see description in the text)	10 × 10	100 × 100	5.0	2	test pattern	0.09 × 0.13	0.5 × 0.5	2.5	2
experiment 4 effect of the tandem mode on a high elemental concentration sample	small region of DGTs #1 to #6 (see description in the text)	60 × 40	100 × 100	5.0	25	small region of mineral sample RB 9B	2.8 × 1.8	1.0 × 1.0	0.3	30
experiment 5 effect of the tandem mode on a low elemental concentration sample	DGT #5 (see description in the text)	141 × 78	100 × 100	2.0	45	one longitudinally sectioned wheat grain	7.0 × 2.9	2.0 × 2.0	0.4	35

DGT gels (described below) were mounted on a large custom-made aluminum frame (108 × 31 cm) using a clear adhesive tape (Figure S2) and analyzed using the upstream large-area scanning “milliprobe” (Figure S3), which can scan objects up to 600 × 1100 mm. Wheat and mineral thin sections were mounted on a 100 × 100 mm sample holder between two pieces of the Ultralene film^26^ and analyzed using the downstream Kirkpatrick–Baez (KB) mirror microprobe. Samples at both scanning stations were analyzed using 384-element Maia detectors in backscatter geometry. An incident flux was typically 1.1 × 10^9^ photons/second for the microprobe station and 1.2 × 10^9^ photons/second for the upstream milliprobe. The beam size was 100 μm on the milliprobe and 2 μm on the microprobe.^25^ All samples were analyzed in “on-the-fly” mode where the horizontal axis is scanned in the continuous motion with discrete vertical steps. The photon energy of the incident X-ray beam was set at 18.5 keV using a Si(111) monochromator. The beam was focused to the desired size using secondary source aperture (SSA) slits for DGT analysis and KB mirrors for downstream specimen analyses. The XFM data were analyzed, and elemental concentrations were quantified using GeoPIXE.^25,27−29^

Air absorption losses to the microprobe were minimized with a modified helium flight tube that had an approximately 79 cm air gap from its upstream window to the milliprobe ion chamber. Photon flux to the milliprobe was controlled by the SSA slits. If the flux was too great for the detection system on the microprobe, the so-called clean up slits, upstream of the KB focusing optics, could be narrowed further for the optimal detector count rate.

### Sample Preparation

#### Downstream Samples: Biological and Mineral Thin Sections

Preparation of the wheat thin sections (210 μm thickness) has been described previously.^26^ The mineral sample (RB 9B) is a lawsonite eclogite from Port Macquarie, eastern Australia, as described previously by Hand et al.,^30^ and from the same locality documented by Tamblyn et al.^31^ It is characterized by a mineral assemblage comprising lawsonite, garnet, omphacite, ferroglaucophane, phengite, and chlorite as the major phases, with abundant accessory zircon and titanite. Mineral thin sections (30 μm thickness) were prepared by first mounting a thick mineral sample (approximately 10 mm) onto a 76 × 25 mm GE fused quartz microscope slide (ProSciTech Pty Ltd) using Araldite GY 191 epoxy resin. The desired sample thickness was achieved by progressive grinding and polishing using Microgrit WCA series aluminum oxide lapping powders, with the final polish achieved on a cloth lap with a 1 μm diamond paste. The sample was then ultrasonicated to remove any surface contamination originating from the sample preparation process.

### Upstream Samples: DGT Gels

#### DGTs Tested in This Experiment

Six DGT-binding gels were tested for their suitability for XFM analysis and for their effect on downstream sample analysis. The six gels were composed of one of three gel matrices; *bis*-acrylamide on the cellulose acetate membrane, polyacrylamide gel, and polyurethane gel. Different binding agents were also used to investigate the 2D visualization of cationic and anionic nutrient diffusion from fertilizers in soil. The following DGT-binding layers were tested: (1) membrane-based *bis*-acrylamide + ferrihydrite (BA-Fe); (2) membrane-based *bis*-acrylamide + Chelex (BA–CH); (3) polyacrylamide + Chelex (PA–CH); (4) polyacrylamide + Chelex–Metsorb (PA–CH–MS); (5) polyurethane + Chelex (PU–CH); and (6) polyurethane + Chelex–Metsorb (PU–CH–MS).

Compared to the most common conventional soil DGTs, which are typically 2.5 cm^2^, the devices used in this project were relatively large (from 80 to 170 cm^2^). Therefore, to prevent the *bis*-acrylamide gels tearing when being handled, these gels (#1 and #2) were prepared on a cellulose acetate membrane brace (0.45 μm pore size, Sterlitech Corporation) for structural integrity. Preparation methods for all gels (binding and diffusive layers) are given in the Supporting Information.

### DGT Deployment in Fertilizer-Amended Soils

#### Setup of Soil Incubation Studies

For all DGT experiments, a pH neutral clayey arable soil (pH_1:5 water_ = 7.4)^20^ collected near Forbes, New South Wales (Australia) was used. Additional soil properties are given in a study by Arias et al.^20^ Field-collected soil was dried at 60 °C for 7 d, ground using a jaw crusher (Bico) and disc mill (Bico), and then passed through a 2 mm sieve. Soil (400 g) was weighed into a 203 × 143 × 53 mm polypropylene container and then brought to 80% of maximum water holding capacity using ultrapure deionized water and homogenized. Six containers were prepared for the six DGTs. Lids were put on the containers, which were then stored in the dark in a temperature-controlled room at 20 °C for 24 h to equilibrate before adding granular fertilizers.

Four granular fertilizers were added to each container (see Figure S4 for positioning of granules in soil); nanoparticulate zinc oxide (ZnO)-coated urea (IcON, Sonic Essentials), microparticulate ZnO-coated urea (Nanosun, Sonic Essentials), a commercial micro- and macronutrient fertilizer (Powerfeed), and a Zn-sulfate (Zn-S) formulation. The Zn-S granules were obtained from a blended monoammonium phosphate (MAP) fertilizer containing 1% Zn (w/w). Noting this was a blended fertilizer and not a co-granulated formulation, we only applied the Zn-containing granules to soil not the MAP granules. Fertilizers were chosen to provide a source of cationic (Zn^2+^) and anionic nutrients (PO_4_^3–^). Each granule was pushed 4 mm below the soil surface and incubated in the lidded container for 28 days at 25 °C. Mass fractions of macro- and micronutrients in fertilizers—measured using ICP–MS following microwave digestion of granules using concentrated (70%) nitric acid—are given in Table S1.

### DGT Assembly and Deployment

DGTs were assembled in four layers: acrylic (1 mm thickness) cut to the size of the binding layer (as a backing support), cellulose acetate membrane (for non-membrane-based DGTs to support the binding layer for XFM analysis), binding layer, and diffusive layer, with all layers held in place by metal clips. The DGT was then inverted (i.e., diffusive layer in contact with the soil surface) and deployed on the soil with the center of the DGT aligned with the central fertilizer granule. To ensure the complete contact between the DGT and the soil surface, plastic wrapping was placed on the acrylic layer and the lid put on the container to act as a piston. After 24 h at 20 °C, the DGT was removed, the diffusive layer discarded, the binding gel removed from the acrylic, and the edges carefully rinsed with ultrapure deionized water to remove any adhered soil particles. The binding layer was then oven-dried in an acrylic frame, to prevent shrinkage and curling, at 40 °C for 10 min (Figure S5).

## Results and Discussion

### XFM Data Acquisition

For tandem scanning measurements, control scan software running in interactive data language (IDL) was used as normal for controlling the microprobe scanning appraratus.^25^ To enable tandem scanning with the milliprobe, another input output controller with uniquely named process variables was used for milliprobe control if, and only if, tandem scanning was requested with scan control software. The milliprobe scans were run from a second IDL session on a separate computer. The microprobe scan was launched first, followed by the milliprobe, with a keyword selecting tandem scanning. The milliprobe tandem scan was started second as it has no control over the radiation protection sample shutters (a true follower). Data were saved into separate directories corresponding to the probe detector.

### Performance of Large DGT-Binding Gels for XFM Analysis (Experiment 1)

#### Optimization of Large DGT-Binding Gels

Given the large size of the DGTs used in this study, binding gels were evaluated for the ease with which they could be prepared for XFM analysis and their suitability for XFM analysis itself. Three optimization parameters were considered: (1) handling properties of the gels, (2) shrinkage and drying effects, and (3) analytical constraints. Considering these parameters, polyurethane gels containing Chelex and Metsorb as binding agents were found to be most favorable for XFM analysis. Polyurethane gels were more elastic and tear-proof than equivalent-sized *bis*-acrylamide gels (which require a membrane support when made at a thickness of 100 μm). Provided the polyurethane gels were dried in the custom acrylic frame, this gel type did not shrink upon drying, in contrast to poly- and *bis*-acrylamide gels. Finally, to avoid the interferences between the binding agent and analyte of interest (phosphate ion), we chose to use titanium-based Metsorb as the anionic-binding agent rather than commonly used zirconium-oxide: Zr has L fluorescence lines (Lα 2.039 keV and Lβ 2.124 keV) that overlap with P Kα (2.014 keV) and Kβ (2.139 keV) energies, and as a result, Zr could not be used as a binding agent. Further discussion of the optimization and handling properties of large DGTs is given in the Supporting Information.

### Mapping the Soil Distribution of Available Cationic and Anionic Plant Nutrients Using XFM Analysis

**Figure 2:**
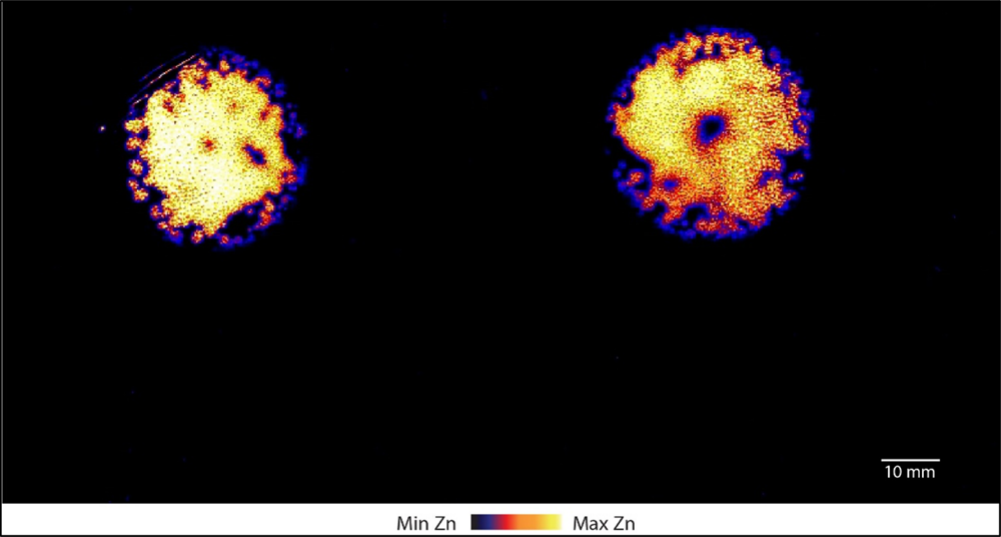
XFM image showing the distribution of Zn in the polyurethane + Chelex-binding gel (PU–CH, gel #5) following DGT deployment for 24 h on fertilizer-amended soil after 28 d incubation. The centers of the zinc hotspots correspond to the location of the “Zn-S” fertilizer granules. A photograph of this gel is shown in Figure S2a.

To the best of our knowledge, this is the first time that XFM analysis has been used to map the distribution of potentially available nutrients in DGT-binding gels. Overall, XFM analysis of DGTs showed that Zn was relatively mobile and potentially available after 4 weeks when applied to soil predominantly as the Zn-S fertilizer (Figure 2)—the dominant phase of Zn in this fertilizer was gunningite (ZnSO_4_·H_2_O), as determined by X-ray diffraction (XRD) (Figure S6), which is readily soluble in water (57.7 g/100 g water at 25 °C).^32^ After 28 d, potentially available Zn diffused approximately 16.5 mm from the Zn-S fertilizer granules. This was best demonstrated by the PU–CH–MS and PU–CH-binding gels where a symmetrical distribution of Zn was observed (Figures 2, S7 and S8). The absence of detectable DGT-Zn in the soil surrounding ZnO-urea granules may have been due to (i) the forms of Zn and their low solubilities, (ii) Zn fertilizer content, (iii) short DGT deployment time, (iv) soil pH effects, and (v) precipitation reactions in the fertosphere, or a combination of all these factors and is further discussed in the Supporting Information.

Phosphorus was not detected in any binding gel that contained anionic-binding agents, that is, ferrihydrite or Metsorb (gels #1, #4, and #6) (images not shown). This may be due to the lower P content in all fertilizer granules (values are given in Table S1) and consequent low mass of P that accumulated in the gels. We believe that analytical constraints also contributed to the lack of detectable P. Phosphorus, being a lighter element, is preferentially detected from shallower parts of the sample compared to Zn.^1^ Given that Metsorb, to which P binds, was distributed throughout the binding layer and not just at the surface, self-absorption may have occurred in the polyurethane matrix (e.g., over 40% of the P fluorescence signal is absorbed by 10 μm of polyurethane). Analysis was also performed in air, not under vacuum, leading to ∼13% absorption of the P K-edge fluorescence when the detector was 2 mm from the sample. The combination of these factors is most likely to have led to the absence of a detectable P signal.

### Effect of Upstream DGT Gel Analysis on Downstream Beam Properties (Experiment 3)

**Figure 3:**
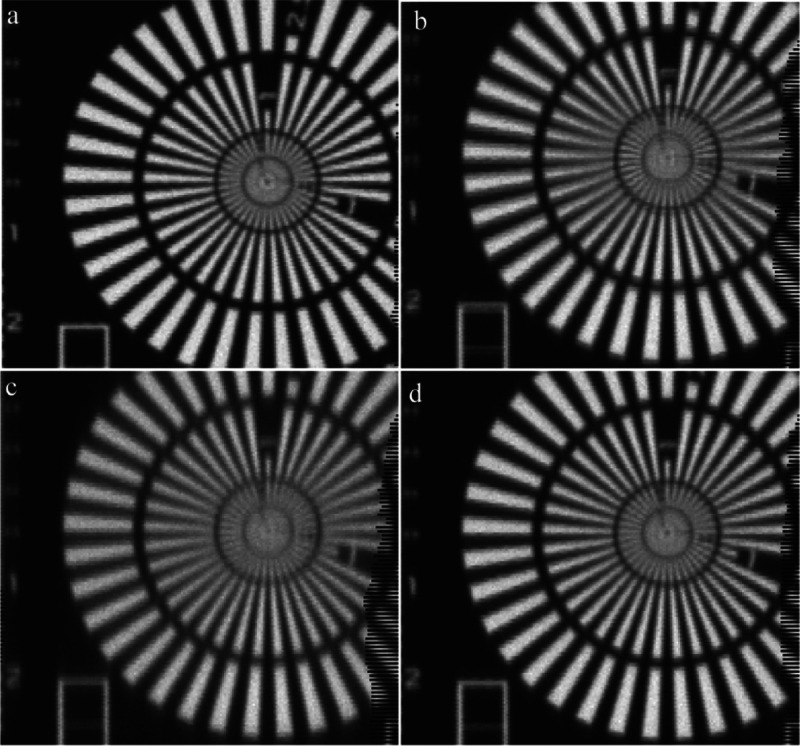
Test patterns mapped on the downstream KB microprobe with the (a) upstream milliprobe detector removed from the beam path and no gel on the milliprobe, and, with the following gels on the milliprobe (b) *bis*-acrylamide (BA–CH), (c) polyacrylamide + Chelex–Metsorb (PA–CH–MS), and (d) polyurethane + Chelex–Metsorb (PU–CH–MS). See Figure S9 for XFM images of test patterns with all gel types.

A test pattern made from two metal layers of 450 nm Au and 60 nm Cr was used to investigate the effect of tandem XFM scanning on beam properties by placing this target downstream of the binding layers (Figure 3). Reduced sharpness of the test pattern was evident when the PA–CH–MS was analyzed upstream (Figure 3c). In fact, this gel also reduced the transmissivity of the beam by the largest extent among all gels tested (12.6% decrease in flux, Table S2).

In contrast, PU had a negligible effect on the microprobe beam focus. However, BA and PA gels appeared to degrade beam focus the most (primarily the vertical focus) (Figure 3b,c). Vertical line profiles of the test pattern (Figure S10) show a significant effect on beam focus when the PA–CH and BA–CH gels were scanned upstream compared to the line profiles without an upstream gel in place and the upstream detector removed, and, compared with PU–CH (Figure S9). The beam focus was less effected in the horizontal direction (Figure S11). Cellulose acetate, on which all gels were mounted, did not affect the beam focus (Figure S9). With the exception of PA–CH–MS, transmittance of the beam through most DGT gels was high (>95%; Table S2). Based on these data, we therefore considered a minimum transmittance of 90% to be acceptable for tandem probe analysis of our samples with these DGT-binding gels. To determine whether transmittance varied during raster scanning, an image of the flux (the transmission ion chamber signal) (Figure S12) was taken from the scans of the polyurethane gels, that is, the binding gels that were selected for their favorable properties. For these analyses, the lower intensity threshold was set to 97, whereas the upper intensity threshold was kept at 100. The results (Figure S12) showed that flux variation during raster scanning was <3% and thus insignificant.

### Effect of Upstream DGT Gel Analysis on Downstream Analysis of a High Elemental Concentration Sample (Experiment 4)

**Figure 4:**
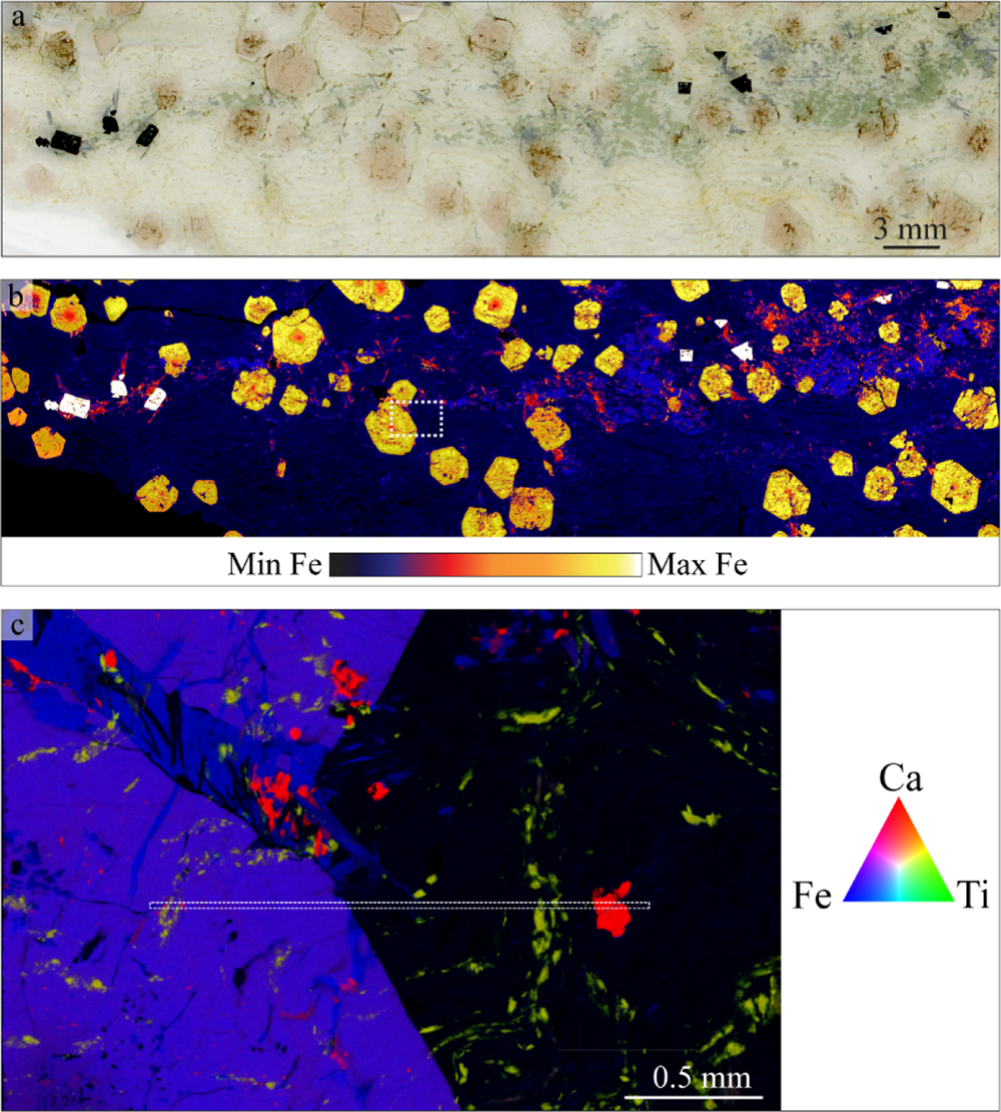
(a) Optical scan of the mineral sample; (b) XFM map showing the distribution of iron in the mineral; (c) XFM map of the region of interest in B (indicated by the white dashed rectangle in (b) showing the elemental distribution of calcium (red), titanium (green), and iron (blue). The XFM map shown in (b) was mapped with a pixel size of 1 μm and (c) was mapped at 3 μm. To evaluate the effects of tandem scanning, data were extracted from within the thin rectangle in (c) and are presented in Figure 5.

The effect of gel type on downstream high-resolution microprobe analysis was evaluated by repeatedly scanning a small (2.8 × 1.8 mm) heterogeneous region of the mineral sample (Figure 4)—selected from the XFM image of the entire mineral thin section (Experiment 2; Figure 4b)—at high resolution (1 μm) while simultaneously scanning each of the DGT-binding layers upstream. The BA gel (gel #2) was not scanned because it was not a promising candidate for future experiments due to poor drying properties.

The XFM map of the entire mineral thin section (Experiment 2), as depicted in Figure 4b, shows that Fe is strongly concentrated in a sulfide phase (pyrite; white hotspots), with progressively lower concentrations in garnet (coarse yellow-orange polygons), ferroglaucophane (fine red shards), and omphacite (purple clusters). Garnet grains also show internal zonation in Fe, with decreasing Fe concentrations from core to rim. A small region of interest was chosen for higher resolution mapping (Experiment 4). The elemental distributions of Ca, Ti, and Fe in this region (Figure 4c) show the arrangement of lawsonite, titanite, garnet, and ferroglaucophane. Lawsonite forms small grains (bright red colors) with no Fe or Ti present, whereas titanite is rich in Ti and Ca with little Fe (yellow-green colors) and forms sigmoidal and anastomosing inclusion trails throughout the sample. Garnet (purple) and ferroglaucophane (blue) both have comparatively high Fe concentrations, with garnet also having an appreciable amount of Ca present.

**Figure 5:**
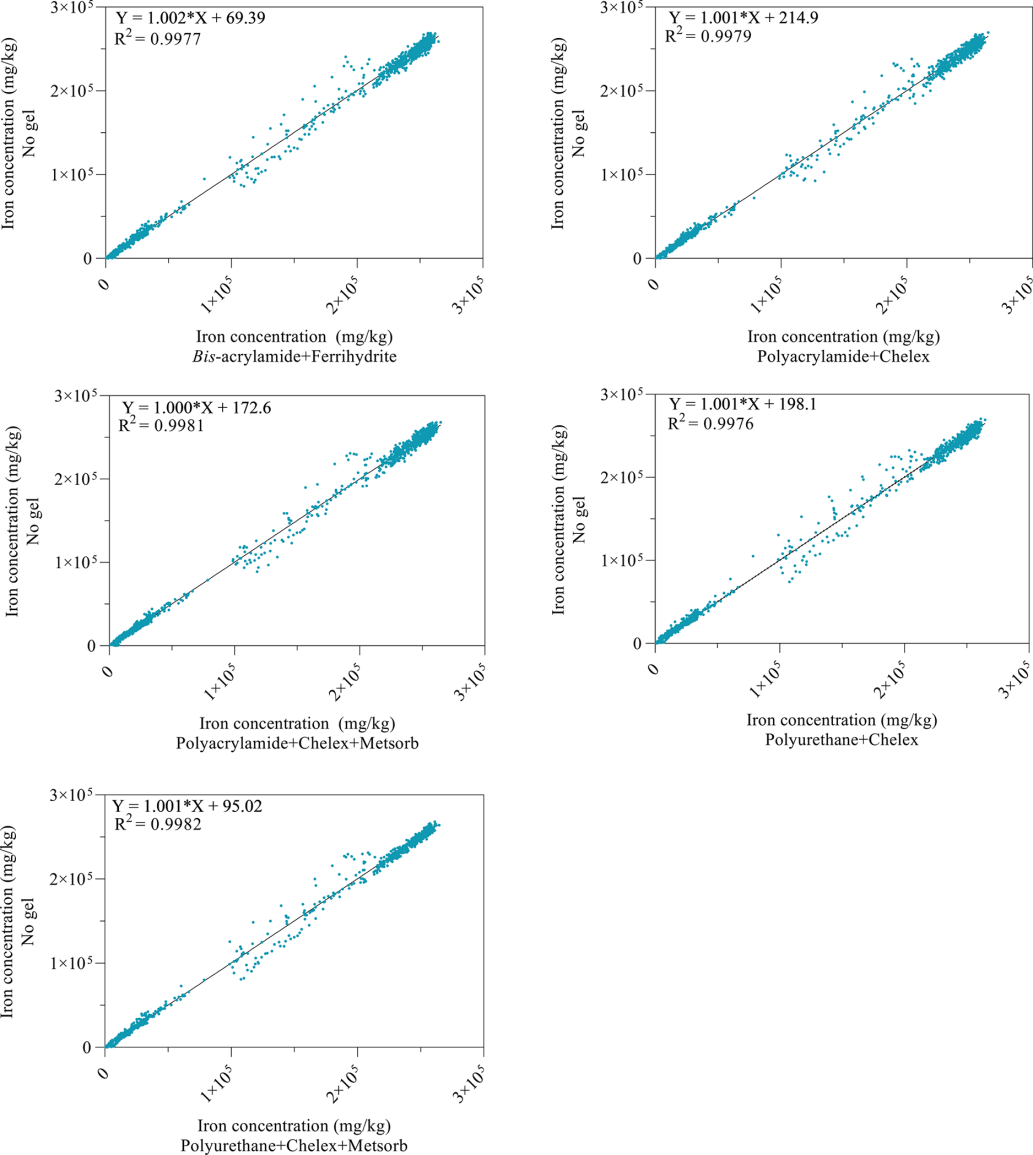
Iron concentrations (mg/kg) measured in the region of interest in the mineral sample, as shown by the white box in Figure 4c. Iron concentrations measured without an upstream gel (*y*-axis) are plotted against those measured while tandem scanning each of the five different gel types upstream (*x*-axis). This mineral sample was mapped with a pixel size of 1.0 μm.

This small region of interest was scanned in tandem with each of the binding gels. The relative concentrations of Ca, Ti, Fe, Y, and Zr were extracted from the area indicated by the dashed rectangle shown in Figure 4c. These data (Figure 5) demonstrate that none of the binding layers appeared to affect the elemental concentrations. Measured values were relatively high; for example, Ca and Fe were in the range 10–20% w/w, Ti was between 5 and 10% w/w, Zr was up to 4% w/w, and Y was distributed in the range 0.01–0.1% w/w. Tandem scanning did not have a pronounced effect on measured elemental concentrations for any of the gels analyzed (Figures 5 and S13). It is important to note that scans of the region, as shown in Figure 4c, were performed at very high resolution. Therefore, very small differences, as observed in Figure 5, between the scans with and without an upstream gel in place could also be caused by scanning reproducibility (i.e., position reproducibility) which is controlled by hardware, for example, motors that drive the sample stage.

Simultaneous XFM imaging of DGTs and microprobe analysis is therefore unlikely to affect the elemental analysis of the downstream sample when the sample has (a) a well-defined crystal structure, (b) a heterogeneous elemental distribution (compared to the test pattern), and (c) high elemental concentrations.

### Effect of Upstream DGT Gel Analysis on Downstream Analysis of Low Elemental Concentration Sample (Experiment 5)

**Figure 6:**
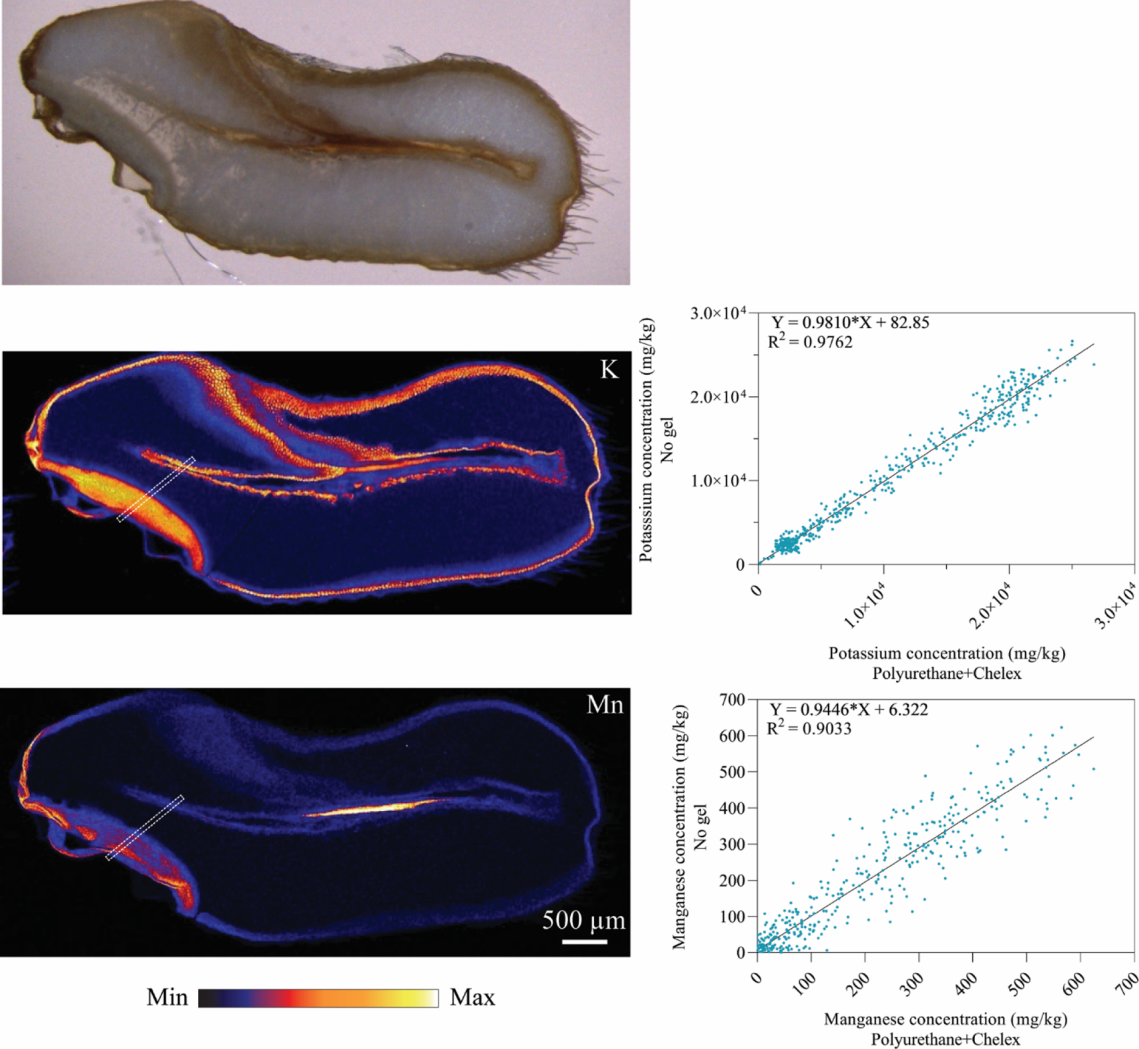
Optical scan of wheat grain longitudinal thin section (top); elemental distribution of potassium (left middle) and manganese (left bottom); and, corresponding elemental concentrations (right) extracted from the dashed rectangular areas in the XFM images. Elemental concentrations without an upstream gel upstream (*y*-axis) are plotted against those with a polyurethane–Chelex gel upstream (*x*-axis). The wheat grain was mapped with a pixel size of 2 μm.

The potential of tandem probe analysis was also evaluated by mapping the distribution of plant nutrients in a wheat grain while simultaneously scanning DGT-binding layers upstream (Figure 6). Before this tandem probe analysis, an overview scan of multiple wheat grain thin sections was performed (Experiment 2), with these XFM images published elsewhere.^26^ For tandem probe analysis, elemental distributions of Cu, Fe, Zn, Mn, and K in wheat grain were first mapped without an upstream gel (single probe mode) and then compared to data collected while simultaneously scanning the PU–CH gel (gel #5); the most promising DGT material. Some peaks of high elemental concentration do not completely overlap when comparing the maps collected with and without the gel (Figure S14). For example, the *R*^2^ value for the correlation between Mn concentrations (Figure 6) measured in the wheat grain with and without an upstream PU–CH gel (*R*^2^ = 0.9033) is lower than that for the equivalent Fe analysis of the mineral sample (*R*^2^ = 0.9976). This is most likely because the wheat sample was removed in-between scans, thereby slightly changing the alignment of the sample with the detector. Similar to the mineral scan, very small differences in the scan position can affect scan reproducibility, which is controlled by hardware. However, the intensity and distribution of signals are very similar as can been seen from the Mn and K concentrations within the traverse section (Figures 6 and S14).

In agreement with previous XFM analysis,^33^ Mn was most strongly localized in the embryo and outer parts of the grain (pericarp and testa) with lower concentrations found in the endosperm. Within the embryo, most Mn was found in the root and shoot primordia. Although at lower concentrations, Mn also accumulated in the layer of aleurone cells of the crease. The region of high Mn concentration in the center of the crease is most likely the pigment strand (a strand of colored tissue at the base of the crease extending its length^34^), which has been shown to accumulate Mn.^33^ As expected, K was found at much higher concentrations than Mn in the grain. However, its pattern of distribution was similar to that of Mn with two exceptions: (1) there was greater accumulation in the scutellum, as shown by the steeper concentration gradient across the embryo to the endosperm and (2) more localization in the aleurone cells of the crease rather than that in the pigment strand. The distribution of Zn in this grain sample has been reported previously.^26^ The data show that simultaneous XFM imaging is unlikely to affect downstream sample analysis of biological samples having low concentrations of heterogeneously distributed elements.

### Advantages and Limitations of Tandem Probe XFM Analysis

Large polyurethane DGT-binding gels (e.g., 10 × 15 cm) can be used to visualize the distribution of potentially available trace elements in soil. These binding gels had superior drying and handling properties compared to *bis*-acrylamide and polyacrylamide gels. The embedded Chelex-binding agent effectively bound potentially available Zn^2+^. However, the distribution of phosphate could not be mapped in this study, most likely due to analytical limitations of XFM, rather than lack of PO_4_^3–^ accumulation in the binding gels that contained anionic-binding groups (i.e., Metsorb or Ferrihydrite). Further experiments are being undertaken to map P distribution under vacuum using XFM and to investigate the efficacy of different Metsorb-containing DGT devices. The simultaneous collection of data from two detectors on the same beamline has previously been demonstrated for small-angle X-ray scattering and wide-angle X-ray scattering^35^ and XFM scanning combined with XRD microscopy.^36^ However, to the best of our knowledge, this is the first time that XFM data have been collected simultaneously from two different samples using two detectors.

Our results show that tandem probe analysis has little effect on the downstream analysis of both high and low elemental concentration samples. Therefore, this configuration can be used to effectively double the output of the XFM beamline by allowing asynchronous sample analysis. As each probe data set is normalized to its own ion chamber, any flux changes (or minor drifts) caused by the DGT sample on the milliprobe are taken into account on the microprobe. While we used Maia detectors for this experiment, any detector type could be used in this configuration. In addition, we have shown that 100 μm thin polyurethane gels have minimal effect on the beam focus compared to *bis*-acrylamide and polyacrylamide gels, which caused beam degradation, as observed at the downstream detector. Therefore, this configuration can be used for future experiments where large polyurethane-binding gels are effectively mapped in the “background” of microprobe sample analysis. Expanding on this, any uniform highly transmissive sample could potentially be used upstream.

There are three main limitations to this new technique. First, as discussed above, the sample upstream needs to be transmissive and homogeneous enough to minimize the beam degradation upstream of the second sample. The choice of an acceptable rate of transmittance would be specific to each experiment and dependent on a number of factors, particularly the elemental concentration of the downstream sample. For our samples, we estimate that a minimum transmittance of 90% is acceptable for tandem probe analysis (based on the beam degradation that was observed for 87% transmittance, Figure 3c). Second, the incident energy is consistent at both detectors during tandem scanning, and a compromise needs to be found between the two samples based on the elements to be investigated. Furthermore, X-ray absorption near-edge structure (XANES) mapping and μ-XANES—two capabilities of this XFM beamline^2,25^ —would not be possible during tandem probe analysis. These techniques are variable-energy techniques where spectra are collected at a number of energies (usually between 60 and 200 different energies) across the absorption edge of an element of interest.^25^ Therefore, these changes in incident energy make XANES imaging and line XANES an impossibility in the tandem probe mode unless identical speciation information was required from identical sized areas from both samples. The third major limitation is logistics. The configuration works best when both samples either have similar scanning times, to allow for concurrent sample changes, or with larger upstream samples and smaller downstream samples mapped at high resolution.

## Conclusions

This is the first time that both scanning stations at the ANSTO XFM beamline have been used to simultaneously map the elemental concentrations of two samples. We have performed successful test experiments using large DGT-binding gels and heterogeneous samples with contrasting matrices and elemental concentrations to demonstrate the potential of this technique. As well as relevance to crop productivity, the new technique is also applicable to environmental contamination studies where different elements can be targeted, and, where DGTs are deployed in other environmental matrices such as waters and sediments. More broadly, we believe that the results can be used to increase the throughput of this and other similar beamlines where any uniform, highly transmissive sample could be analyzed upstream in the “background” of downstream samples. Thus, our approach can benefit researchers from a wide range of fields who are limited by beamline availability. In addition, a similar approach could also be used at synchrotron X-ray absorption spectroscopy beamlines. At these beamlines, reference foil samples are routinely analyzed (in transmission), for energy calibration purposes, downstream of samples. However, it could be possible to analyze two sets of samples (if highly transmissible) in tandem using fluorescence detectors before the analysis of reference metal foils. This would again result in a doubling of sample throughput.

## Supporting Information Available

Additional experimental details, materials and methods for DGT preparation, evaluation of DGT handling properties and fertilizer diffusion in soil, fertilizer composition, beam transmittance data, photographs of the XFM beamline and mounted DGT-binding gels, additional XFM analysis of the test pattern, and quantification of elemental concentrations in mineral thin section and wheat grain using XFM analysis (PDF)
