# Supplementary material for: Tandem Probe Analysis Mode for Synchrotron XFM: Doubling Throughput Capacity
Source: Anal Chem. 2022 Mar 11;94(11):4584–93. doi: 10.1021/acs.analchem.1c04255 (PMC8943523; doi:10.1021/acs.analchem.1c04255)
Supplement: Supplementary file 1 — ac1c04255_si_001.pdf [file ac1c04255_si_001.pdf]

Supporting Information

Tandem probe analysis mode for synchrotron XFM: doubling throughput capacity

Casey L. Doolette,[1] Daryl L. Howard,[2] Nader Afshar,[2] Cameron M. Kewish,[2,3] David J. Paterson,[2] Jianyin Huang,[1,4] Stefan Wagner,[5,6,7] Jakob Santner,[8] Walter W. Wenzel,[7] Tom Raimondo,[1,4] Alexander T. De Vries Van Leeuwen,[4] Lei (Helen) Hou,[1,9] Frederik van der Bom,[9] Zhe (Han) Weng,[9] Peter M. Kopittke[9] and Enzo Lombi[1,4]*

[1]University of South Australia, Future Industries Institutes, Mawson Lakes, South Australia 5095, Australia

[2]Australian Synchrotron, ANSTO Clayton, Victoria, 3168, Australia

[3]Department of Chemistry and Physics, School of Molecular Sciences, La Trobe University, Melbourne, Victoria 3086, Australia

[4]University of South Australia, UniSA STEM, Mawson Lakes, South Australia 5095, Australia

[5]Chair of General and Analytical Chemistry, Montanuniversität Leoben, 8700 Leoben, Austria

[6]Institute of Analytical Chemistry, University of Natural Resources and Life Sciences Vienna, 3430 Tulln, Austria

[7]Institute of Soil Research, University of Natural Resources and Life Sciences Vienna, 3430 Tulln, Austria

[8]Institute of Agronomy, University of Natural Resources and Life Sciences Vienna, 3430 Tulln, Austria

[9]The University of Queensland, School of Agriculture and Food Sciences, St Lucia, Queensland, 4072, Australia

This file contains:

- Additional experimental details, materials, and methods for DGT preparation
- Further evaluation of the handling properties of DGTs
- Distribution of potentially available zinc using DGT XFM analysis

Table S1. Elemental composition of fertilisers.

Table S2. Transmittance of beam through all DGT gels.

Figure S1. XFM beamline at the Australian Synchrotron

Figure S2. DGT binding gels mounted in the upstream position on the XFM beamline

Figure S3. DGT binding gels mounted on aluminium frame with transparent tape for XFM analysis

Figure S4. Placement of fertiliser granules in soil

Figure S5. DGT binding gel drying in acrylic frame

Figure S6. XRD pattern of "Zn-S" granules used in the DGT experiment

Figure S7. XFM images of binding gels #1, #2 and #5 showing Compton scatter and zinc distribution (Experiment 1).

Figure S8. XFM images of binding gels #3, #4 and #6 showing Compton scatter and zinc distribution (Experiment 1).

Figure S9. Test patterns mapped on the downstream KB microprobe while scanning gels upstream on the millprobe Maia detector.

Figure S10. Vertical line profiles of test pattern scans

Figure S11. Horizontal line profiles of test pattern scans

Figure S12. XFM image of polyurethane gels showing flux

Figure S13. Concentrations (mg/kg) of calcium (A), titanium (B), iron (C), yttrium (D) and zirconium (E) in the region of interest in the mineral sample (Figure 4c)

Figure S14. Optical scan of wheat grain longitudinal thin-section with elemental distributions of potassium and manganese

Preparation of DGTs

Reagents and solutions

Analytical reagent grade chemicals and ultrapure deionised water (18.2 MΩ cm, Milli-Q Advantage 10, Millipore) were used in the preparation of all DGTs. All binding gels (#1 to #6) were stored in acid-washed polypropylene containers, containing ultrapure deionised water prior to use.

Bis-acrylamide binding gels (1 and 2)

The ferrihydrite binding gel (#1) was prepared by *in situ* precipitation of ferrihydrite within the hydrogel according to Luo et al.[31] with minor modifications. Briefly, membrane-based *bis* acrylamide diffusive gel sheets were immersed in a solution of 40.04 g of $Fe(NO_3)_3{\cdot}9H_20$ (Sigma-Aldrich) dissolved in 1 L of ultrapure deionsed water. After 2 h of immersion, the gels were removed, rinsed with ultrapure deionised water then immersed in 1 L of a 0.05 mol L^{-1} MES (2-(*N*-morpholino)ethanesulfonic acid) monohydrate (Sigma Aldrich) buffer solution that had been pre-adjusted to pH 6.7 using approximately 35 mL of 1 mol L^{-1} NaOH (Chem Supply). After 40 min of immersion, and gentle agitation every 10 min to ensure homogenous precipitation of Fe^{3+}, the gels were rinsed three times with ultrapure deionised water before use.

The iminodiacetate-based Chelex®-100 (BioRad) binding gel (#2) was prepared according to Davison and Zhang with the minor modification that we used *bis*-acrylamide membrane based gels instead of polyacrylamide.[32] Gels #1 and #2 were 10 cm × 17 cm.

Polyacrylamide binding gels (3 and 4)

The polyacrylamide-Chelex®-100 (BioRad) binding gel (#3, without a membrane brace) was prepared according to Davison and Zhang.[32] The polyacrylamide-Chelex®-Metsorb® mixed binding gel (#4, without membrane brace) was prepared according to Panther et al.,[33] where Metsorb® (Graver Technologies) is a titanium dioxide-based anion binding agent. Gels #3 and #4 were 8 cm × 10 cm.

Polyurethane binding gels (5 and 6)

Polyurethane-based 100 µm-thin binding gels were prepared using established knife-coating procedures.[34-36] The ether-based hydrophilic urethane material (HydroMed™ D4, Advan Source biomaterials, MA, USA) was pre-cleaned by washing in ultrapure deionised water (1:15, w/v) for one week with four successive water changes and was then used to prepare the PU gel stock solution according to Kreuzeder et al.[35]

The Chelex® 100 (75-37 µm particle size, sodium form; Sigma-Aldrich) and Metsorb® (50 µm particle size; Graver Technologies) binding phases were dry-ground in a planetary ball mill (PM 100, Retsch GmbH, DE) to obtain a particle size ≤10 µm allowing for high resolution solute sampling. Prior to milling, the Chelex®-100 was lyophilised at -55°C and 0.22 mbar (Alpha 1-4 LDplus, Christ, DE). The Metsorb® was ground directly. In total, 2.0 g of dry Chelex® 100 or Metsorb® were added to an acid-cleaned ZrO_2 milling jar (50 mL; Retsch) filled with ten acid-cleaned ZrO_2 milling balls (diameter of 10 mm; Retsch). The resins were milled in five sequential intervals at 550 rpm for 3 min followed by a 20 s break. One gram of the ground Chelex®-100 or ground Metsorb® was added to 10 mL of ultrapure deionised water, vigorously mixed by hand, sonicated for 10 min, and fixed in an overhead shaker for 72 h at 10 rpm to obtain a 10% (w/v) resin ultrapure deionised water

suspension for the Chelex®-100 and Metsorb®. For PU-CH gels, 1.5 mL of the CH suspension was mixed with 13.5 mL of the PU gel stock solution, and for PU-CH-MS gels, 1.5 mL of each CH and MS suspension were mixed with 12 mL of the PU gel stock solution. The resulting PU-based gel suspensions were fixed in an overhead shaker for 2 h at 5 rpm to remove any air-bubbles and then knife-coated (single bar 6'', 0.5 mils; BYK Gardner) onto a glass plate equipped with 0.25 mm-thick spacer strips as detailed previously.[35] Gel #5 was 10 cm × 15 cm and Gel #6 was 12 cm × 8 cm.

Diffusive layer

For DGTs #1 to #4, a *bis*-acrylamide membrane-based gel was used as the diffusive layer (200 µm thickness). These gels were prepared as previously described[20] with minor modifications. Specifically, diffusive gels were prepared on pre-cut cellulose acetate filter membranes (0.45 µm, 60-100 µm thickness, Sterlitech Corporation) instead of a polyethersulfone (PES) membrane. For the polyurethane DGTs (gels #5 and #6), the diffusive layer was a cellulose acetate membrane.

Handling properties of DGTs

Handling properties

Of the three gel materials tested (polyurethane, polyacrylamide and *bis*-acrlylamide), polyurethane-based binding layers had the optimal handling properties. Polyurethane did not curl during manual manipulation, it was relatively tear-proof, and very thin binding layers could be prepared without the need for a membrane support. Comparing the two acrylamide-based gels, *bis*-acrylamide membrane braced gels were superior to polyacrylamide as the latter were difficult to handle due to their high degree of elasticity which caused curling.

Handling properties of the binding layers are primarily determined by the matrix material. Although polyurethane had the best handling properties, the most commonly used gel matrix in DGT devices is polyacrylamide[37] where it is used as both the diffusive and binding layer. The polyacrylamide polymer is comprised of acrylamide subunits and can be prepared as a linear structure or as a cross-linked polymer. Polyacrylamide can be cross-linked using an agarose derivative (termed APA gel) or *bis*-acrylamide at 5%,[15] with the latter having a smaller pore size.[38] Elasticity, or conversely, stiffness, can be adjusted by changing the concentration of *bis*-acryalmide cross-linker in the polymer.[39] While this elasticity generally makes handling small gels easier (i.e. commercial DGTs), it can present challenges when trying to flatten large gels for deployment – as was observed here – and when slicing gels for analysis. Therefore *bis*-acrylamide gels have been developed as a less elastic alternative although they are more fragile.

Kreuzeder et al.[35] were the first to use polyurethane in DGT devices. The authors developed this technique to overcome problems associated with preparing a Zr-hydroxide polyacrylamide gel for measuring phosphate: when Zr-hydroxide was added to the acrylamide solution it caused instantaneous polymerisation, resulting in a highly heterogonous Zr-hydroxide gel. Substituting a polyacrylamide gel for a polyurethane gel overcame this problem. The polyurethane binding layer is an ether-based hydrophilic urethane polymer which does not require UV curing or a polymerization reaction and instead forms during solvent evaporation.[35] The main advantage of the urethane is that very thin layers can be prepared (100 µm) which are stronger and more tear-proof than equivalent sized *bis*-acrylamide gels (which require a membrane support when made at this thickness). Our results on large DGT devices are in agreement with these observations, where the handling properties of polyurethane based binding layers were superior to those of poly- or *bis*-acrylamide gels.

Shrinkage and drying effects

Of the three materials tested, polyurethane and polyacrylamide exhibited the least shrinkage and curling, and therefore had the most favourable drying properties. The polyacrylamide and *bis*-acrylamide binding gels shrank upon drying, causing small holes to form in some of these gels. Prior to XFM analysis, DGT binding layers must be dried. Drying is necessary for increasing relative elemental concentrations, reducing scattering during analysis, but also for increasing the signal detected from lighter elements (e.g. P), which have lower energy fluorescent X-rays that are absorbed by water. However, drying can cause gel shrinkage.

Smaller binding layers could be dried using a vacuum gel dryer which is commonly used for drying electrophoretic gels. However, as our binding gels were large (10 cm × 17 cm) we used custom-made acrylic frames to dry all binding gels (Figure S5). Each binding gel was first placed on a piece of cellulose acetate membrane, then the

edges (3 mm) of both layers sandwiched between two identical sized polycarbonate frames. For the *bis*-acrylamide (BA) gels, the main issue was that they did not dry flat, which compromised the XFM analyses. And for the BA-CH gel, holes formed throughout the gel upon drying (Figure S2a). Polyurethane gels did not shrink upon drying, provided they were dried in the custom frame.

Analytical constraints

Analytical constraints also require consideration when optimising DGT gels for XFM analysis. For example, interferences between the binding agent and specific analyte of interest must be avoided. We originally proposed to use Zr-oxide as a binding agent for phosphate given its high binding capacity. However, Zr has L fluorescence lines (L*α* 2.039 keV and *L*β 2.124 keV) that overlap with P Kα (2.014 keV) and Kβ (2.139 keV) energies, and as a result Zr could not be used as a binding agent. Therefore, we instead used titanium-based Metsorb® as the binding agent.

As with all XFM analyses, sample thickness is also an important consideration. As discussed by Kopittke et al.[1] lighter elements, such as P, are relatively undetectable from deeper parts of a sample whereas heavier elements like Zn can be detected from the entire sample depth. Therefore, more reliable and accurate elemental distribution maps are produced from thinner gels (i.e. ≤200 µm).

Further discussion of the distribution of potentially available nutrients using XFM analysis of DGTs

Mobility of zinc: a cationic plant micronutrient

The absence of detectable DGT-Zn in the soil surrounding ZnO-urea granules and the commercial fertilisers may have been due to a number of factors. For example, it could be due to the low Zn mass fraction of these fertilisers compared to Zn-S (ZnO-coated urea ~1% Zn; commercial fertiliser ~0.2% Zn vs. Zn-S ~30% Zn). Therefore, even if Zn from ZnO-coated urea did diffuse away from the granule, the total mass of Zn that accumulated on the binding gel may have been below the detection limit of XFM. One way to overcome this may be to deploy DGTs for longer periods of time, allowing for a greater accumulation of Zn in the binding gel and that way improving detection of Zn by virtue of higher gel Zn concentrations.

When Zn is applied to soil in granular form, as was the case for our study, the granule must first dissolve before nutrients can diffuse into the soil. As determined by X-ray diffraction (Figure S6), the dominant phase of Zn in the Zn-S granules was gunningite [$ZnSO_4 \cdot H_2O$] which is readily soluble in water (57.7 g/100 g water at 25°C[40]) whereas ZnO is virtually insoluble.[40] Therefore, it is not surprising that Zn from Zn-S appeared more mobile than ZnO-coated urea. In addition, when urea dissolves in soil it has an alkalifying effect on the fertosphere (i.e. the sphere of soil surrounding the granule).[41] This alkalinity can then induce various sorption and precipitation reactions of Zn in soil which limit the mobility,[42] and thus availability, of Zn as was observed in our study. Such reactions occur less when the dissolving fertiliser (such as MAP[42]) has an acidifying effect.

Few studies that have investigated the diffusion of Zn away from Zn containing fertiliser granules. Those that have have typically used co-granulated fomulations where Zn is present with other nutrients such as P or N. For example, using synchrotron-based µ-XRF analysis, Hettiararchchi et al.[42, 43] showed that ~85% of the Zn in a co-granulated MAP fertiliser granule (MAP+Zn) (9% P, 1% Zn, w/w) remained in the granule after five weeks of incubation in an alkaline calcareous sandy loam (pH_{H_2O} = 8.5). For the ~15% of Zn that did dissolve and diffuse out of the granule, it was retained within 4 mm from the granule. The authors attributed this low level of diffusion to the formation of sparingly soluble Zn-phosphates which restricts Zn solubility under alkaline conditions.[43] Using a visualisation technique, Degryse et al.[44] also showed that Zn diffusion is restricted when Zn is applied to soil with P i.e. as Zn-coated MAP and diammonium phosphate (DAP).

Table S1. Elemental composition of fertilisers.

	% w/w				μg/g							
Fertiliser	Zn	P	S	K	Na	Mg	Al	Mn	Fe	Co	Ni	Cu
Nanoparticulate ZnO-coated urea	0.7	0.004	0.03	0.03	266	384	672	3	83	<LOD	1	3
Microparticulate ZnO-coated urea	0.9	0.003	0.03	0.04	98	364	118	2	84	<LOD	1	19
Commercial (Powerfeed ®)	0.2	1.64	18.44	20.18	9421	6285	694	246	1741	3	5	37
Zn-S	33.0	0.1	15.2	0.1	7032	356	343	604	252	4	5	7

<LOD, concentration of analyte was below the limit of ICP-MS detection

Table S2. Transmittance of the beam through all DGT gel samples. The microprobe (KB) ion chamber (downstream of the milliprobe) was used to measure the incident flux as affected by upstream DGT analysis.

Sample analysed on the upstream milliprobe Maia detector	Microprobe ion chamber, CPS	Transmittance relative to the scan without the milliprobe Maia detector and no DGT
No upstream milliprobe Maia detector, no DGT	222130	1.000
Upstream milliprobe Maia detector in, no DGT	220219	0.991 ± 0.020
Cellulose acetate membrane only	218749	0.985 ± 0.020
BA-Ferrihydrite	220039	0.991 ± 0.020
BA-Chelex	211839	0.954 ± 0.019
PA-Chelex	215179	0.969 ± 0.019
PA-Chelex-Metsorb	194129	0.874 ± 0.017
PU-Chelex	225579	1.016 ± 0.020
PU-Chelex-Metsorb	221769	0.998 ± 0.020
PU-Chelex upstream (mounted on transparency film not cellulose acetate)	218640	0.984 ± 0.020

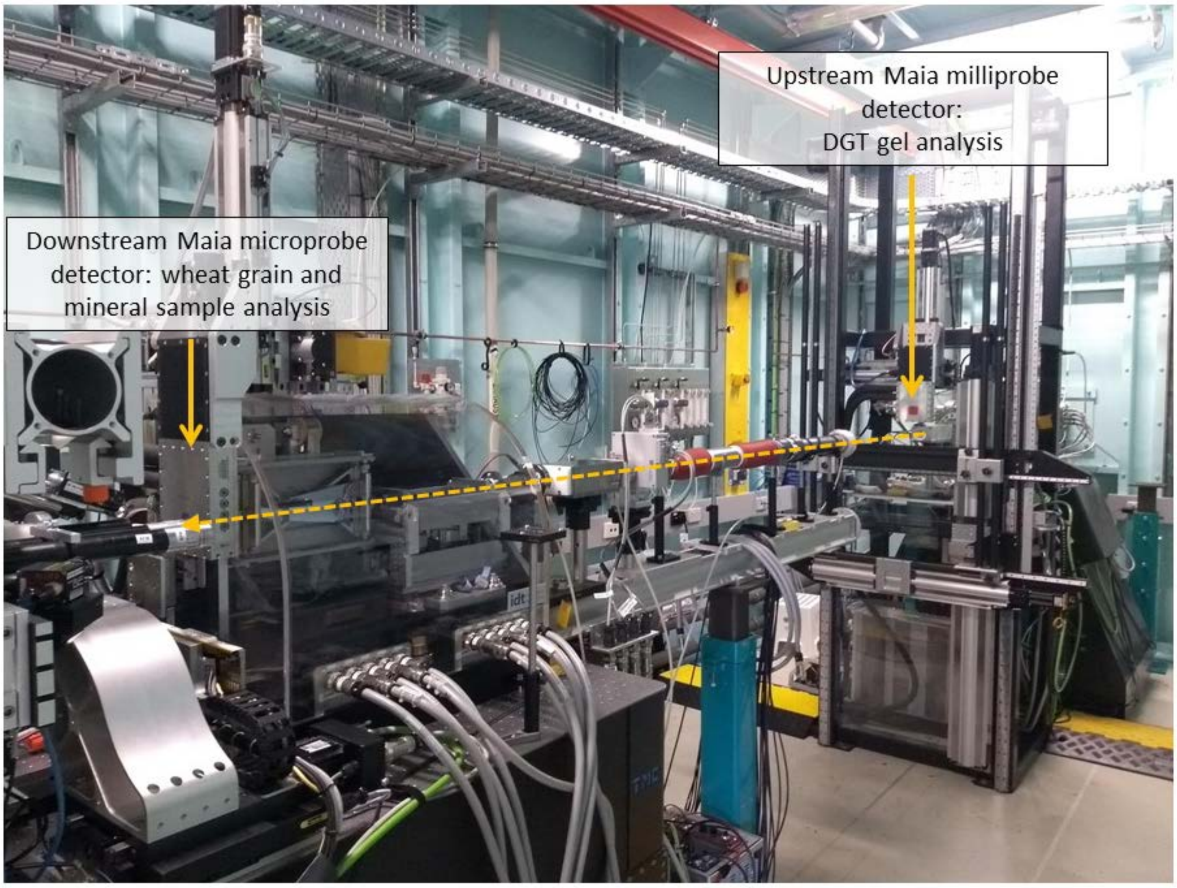

Figure S1: XFM beamline at the Australian Synchrotron. The direction of the beam is indicated by the yellow dashed line.

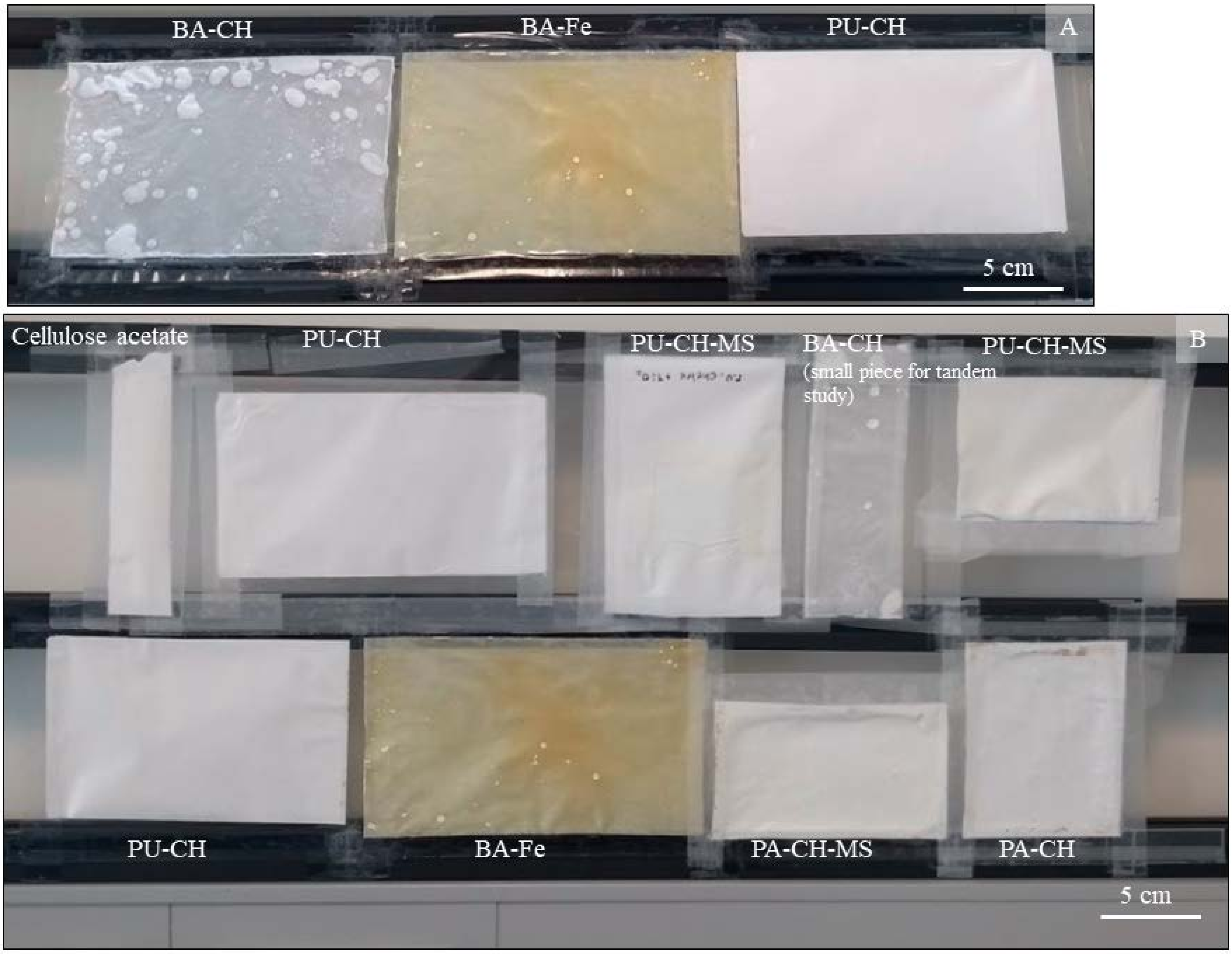

Figure S2. DGT binding gels mounted on aluminium frame with transparent tape for XFM analysis (Experiment 1). The gels in panel (a) were analysed in one XFM scan according to the parameters given in Table 1 of the main text. Gels in panel (b) were scanned using the same XFM scanning parameters except they were scanned individually and therefore the size of the scanned area also differed. All six binding gels are shown: (1) membrane-based *bis*-acrylamide + ferrihydrite (BA-Fe); (2) membrane-based *bis*-acrylamide + Chelex (BA-CH); (3) polyacrylamide + Chelex (PA-CH); (4) polyacrylamide + Chelex–Metsorb (PA-CH-MS); (5) polyurethane + Chelex (PU-CH) (6) polyurethane + Chelex–Metsorb (PU-CH-MS). Replicates are shown for some gels.

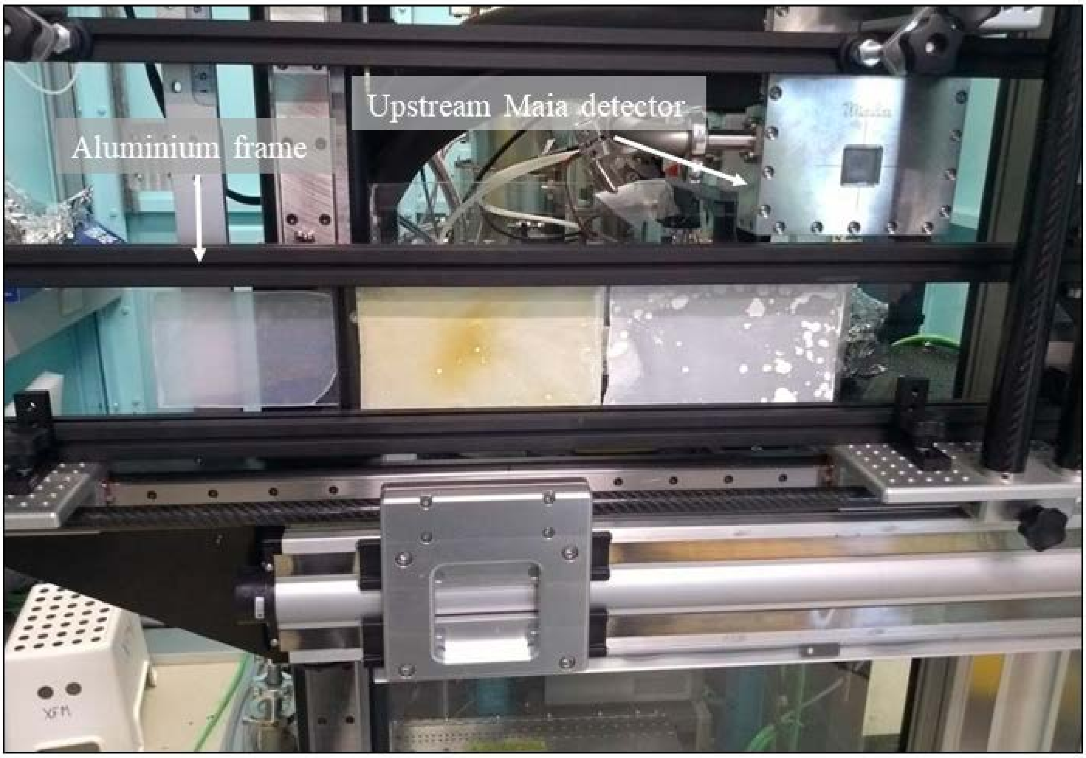

Figure S3: Three binding gels [from left to right: PU-CH-MS on Perspex backing (this was not used for further analysis), *bis*-acrylamide-Fe and *bis*-acrylamide-Chelex] mounted on aluminium frame in the upstream position on the XFM beamline. The upstream Maia detector is also shown.

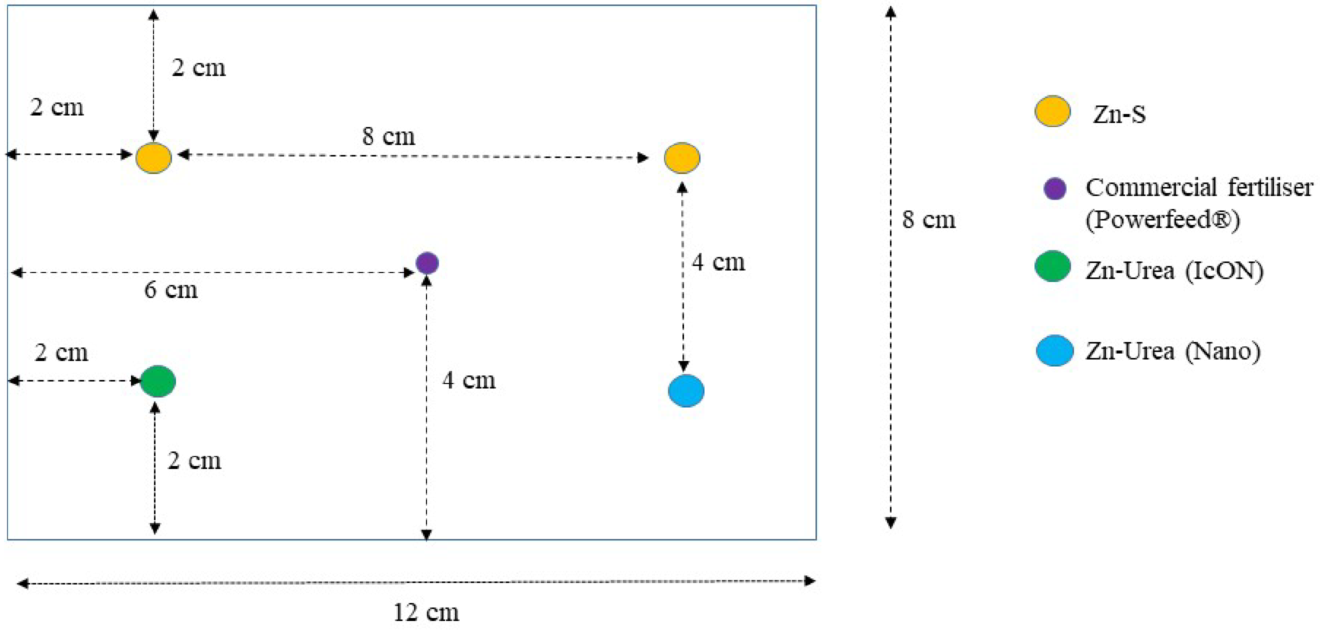

Figure S4: Placement of granular fertilisers in soil.

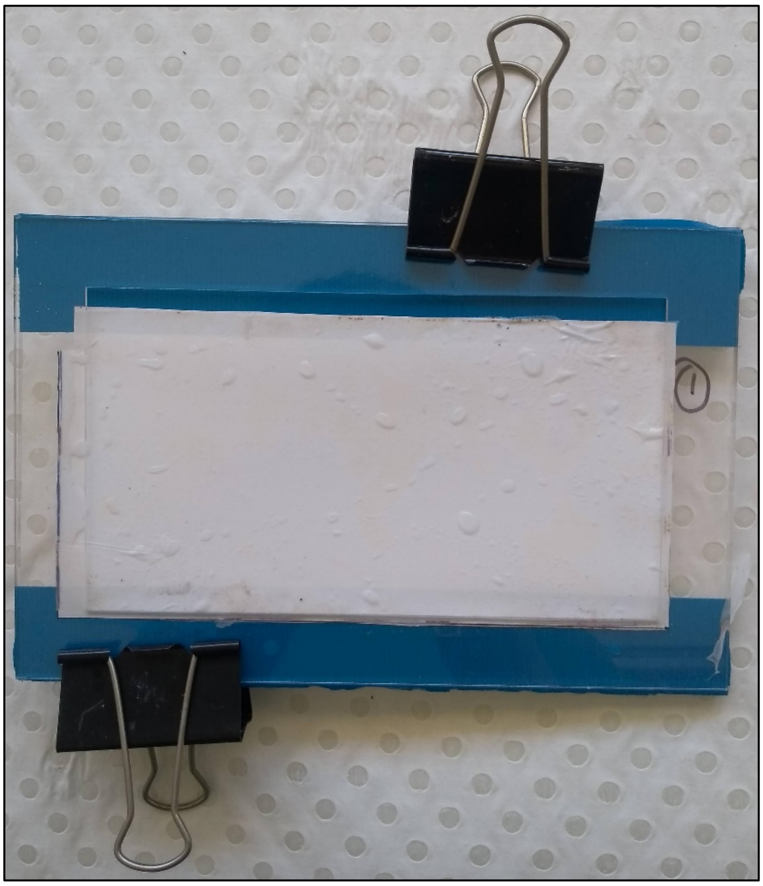

Figure S5: *Bis*-acrylamide-ferrihydrite DGT binding layer (with cellulose acetate membrane) drying in acrylic frame. Water droplets are visible on the binding layer surface.

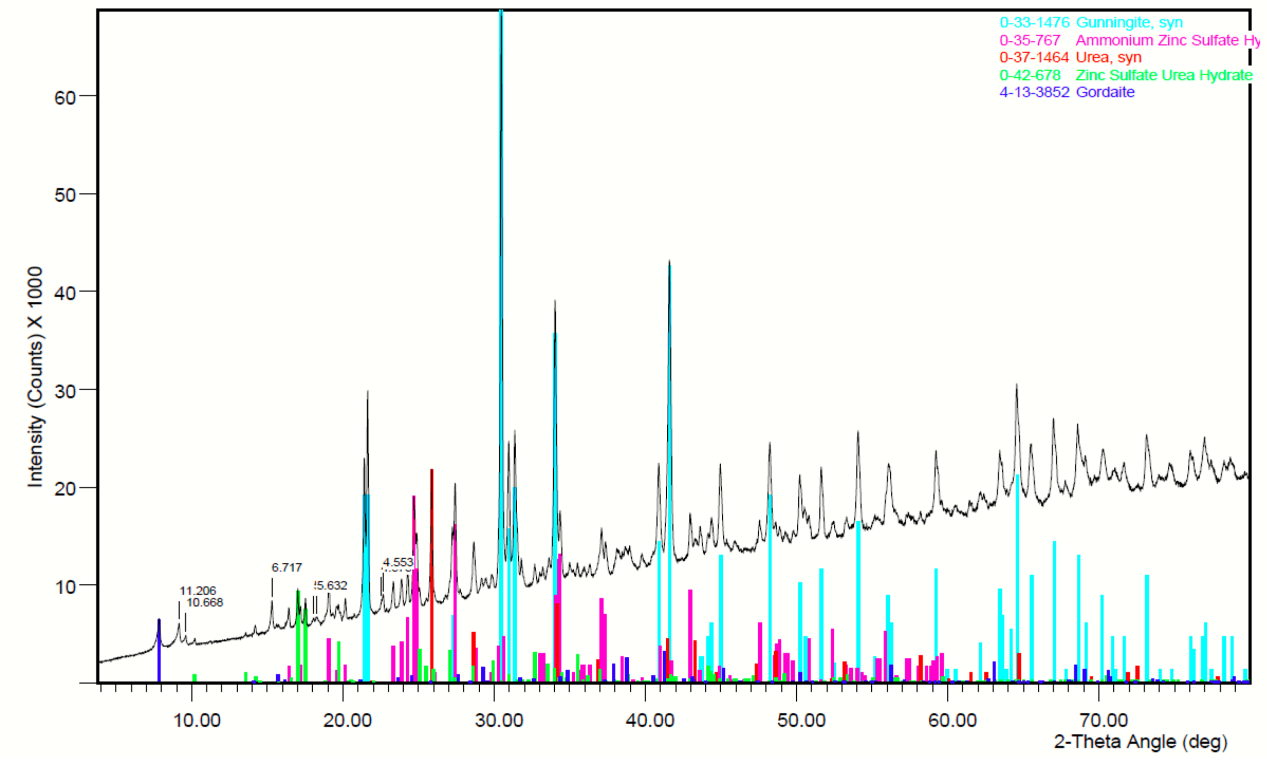

Figure S6: XRD pattern of “Zn-S” granules used in the DGT experiment. Granules were ground to a fine powder using an agate mortar and pestle. The XRD data were collected on a PANalytical X’Pert Pro diffractometer using Fe-filtered Co radiation and an automatic divergence slit. The colour coded stick patterns show the expected positions and intensities for the phase listed in the top right corner of the figure. The labels above the peaks give the d-spacing of peaks that have not been matched to a phase.

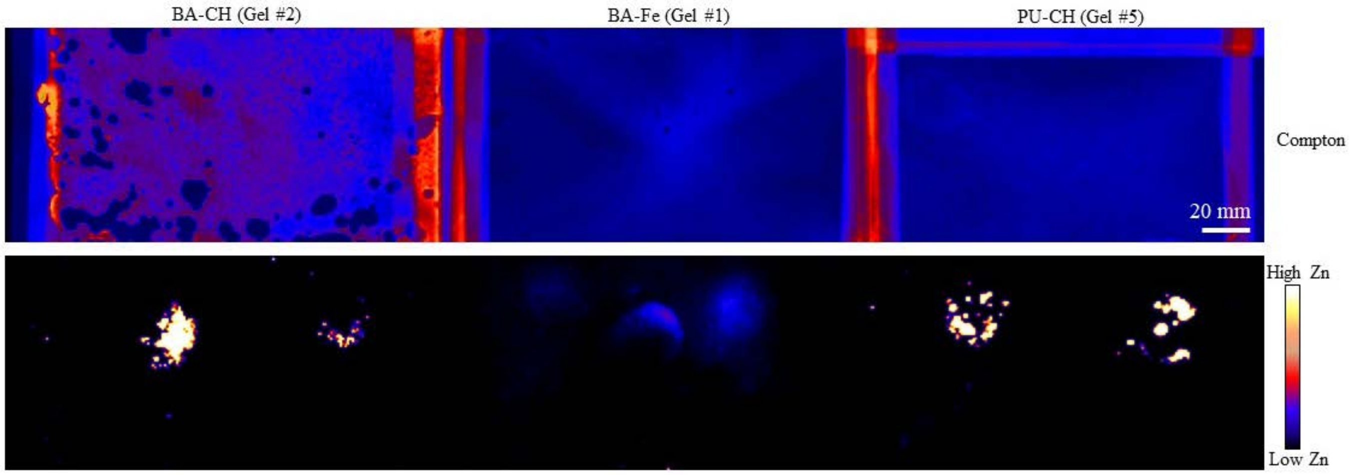

Figure S7: XFM images of binding gels showing Compton scatter (top) and zinc distribution (bottom) from Experiment 1. The XFM scanning parameters are given in Table 1 of the main text. Photographs of the binding gels are shown in Figure S2.

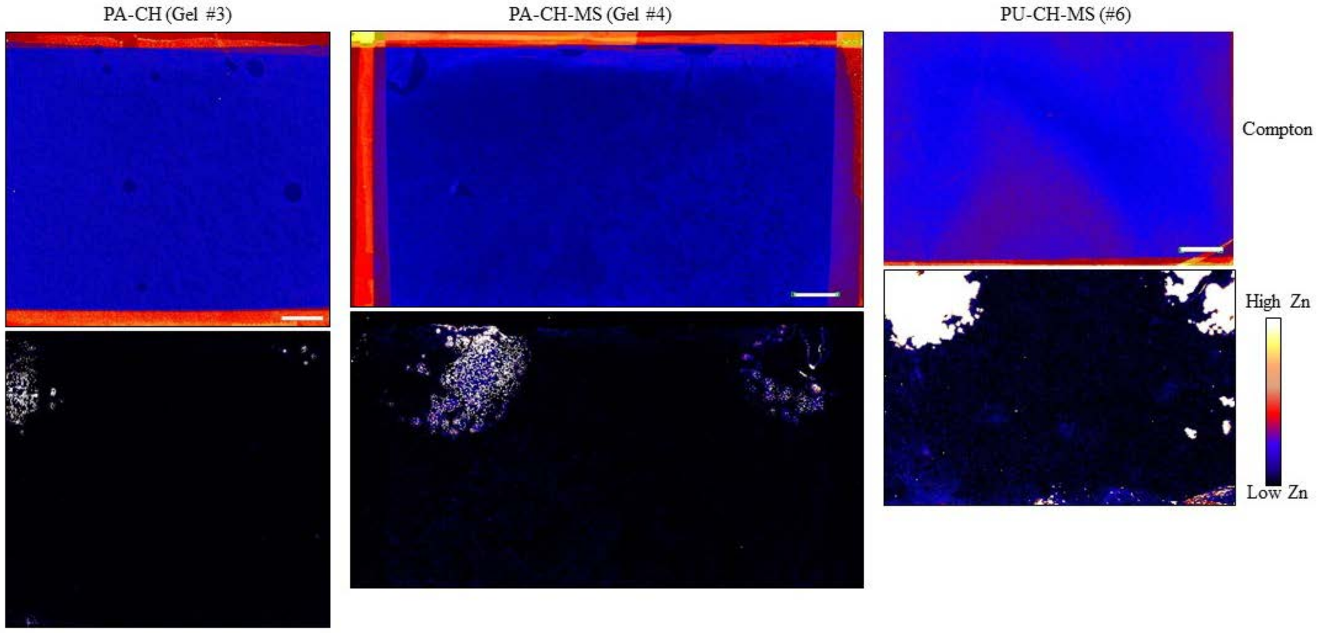

Figure S8: XFM images of binding gels showing Compton scatter (top) and zinc distribution (bottom) from Experiment 1. Each scale bar is equivalent to 10 mm. The XFM scanning parameters are given in Table 1 of the main text. Photographs of the binding gels are shown in Figure S2.

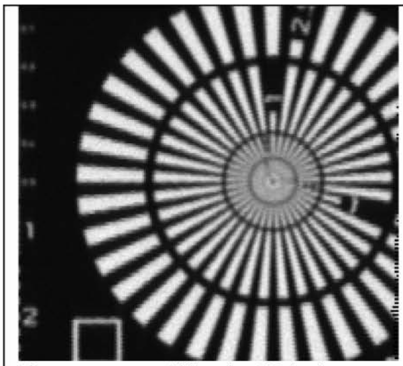

No upstream milliprobe Maia detector, no DGT

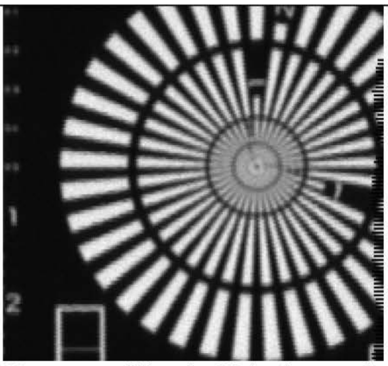

Upstream milliprobe Maia detector in place, no DGT

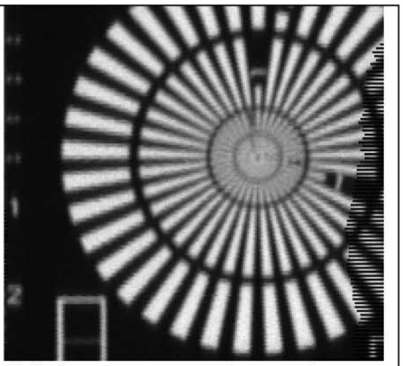

Cellulose acetate membrane only

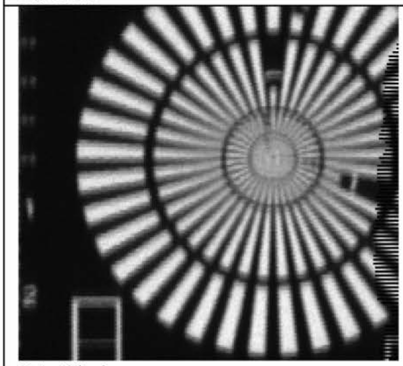

BA-Chelex

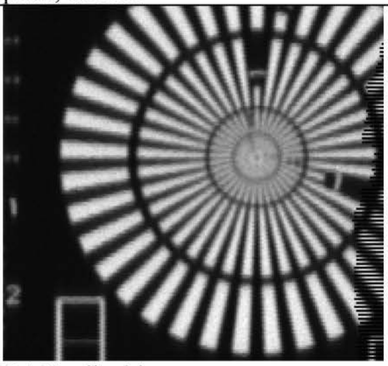

BA-Ferrihydrite

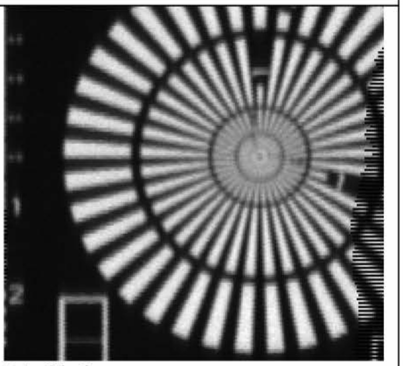

PA-Chelex

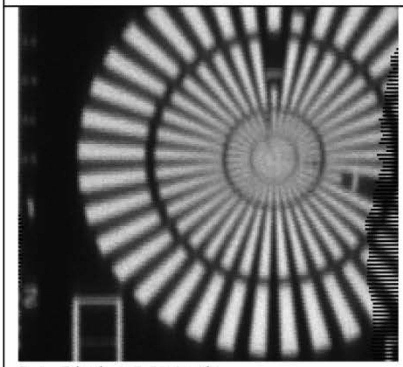

PA-Chelex-Metsorb

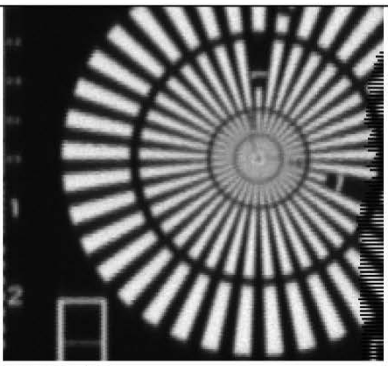

PU-Chelex

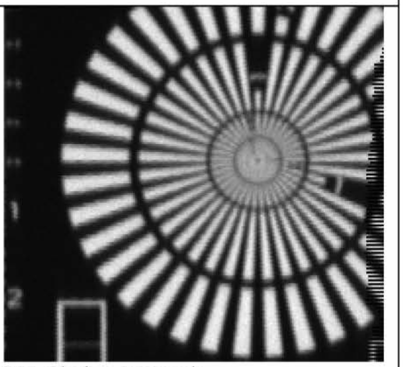

PU-Chelex-Metsorb

Figure S9. Test patterns mapped on the downstream KB microprobe while scanning various gels (shown underneath each test pattern) upstream on the millprobe Maia detector.

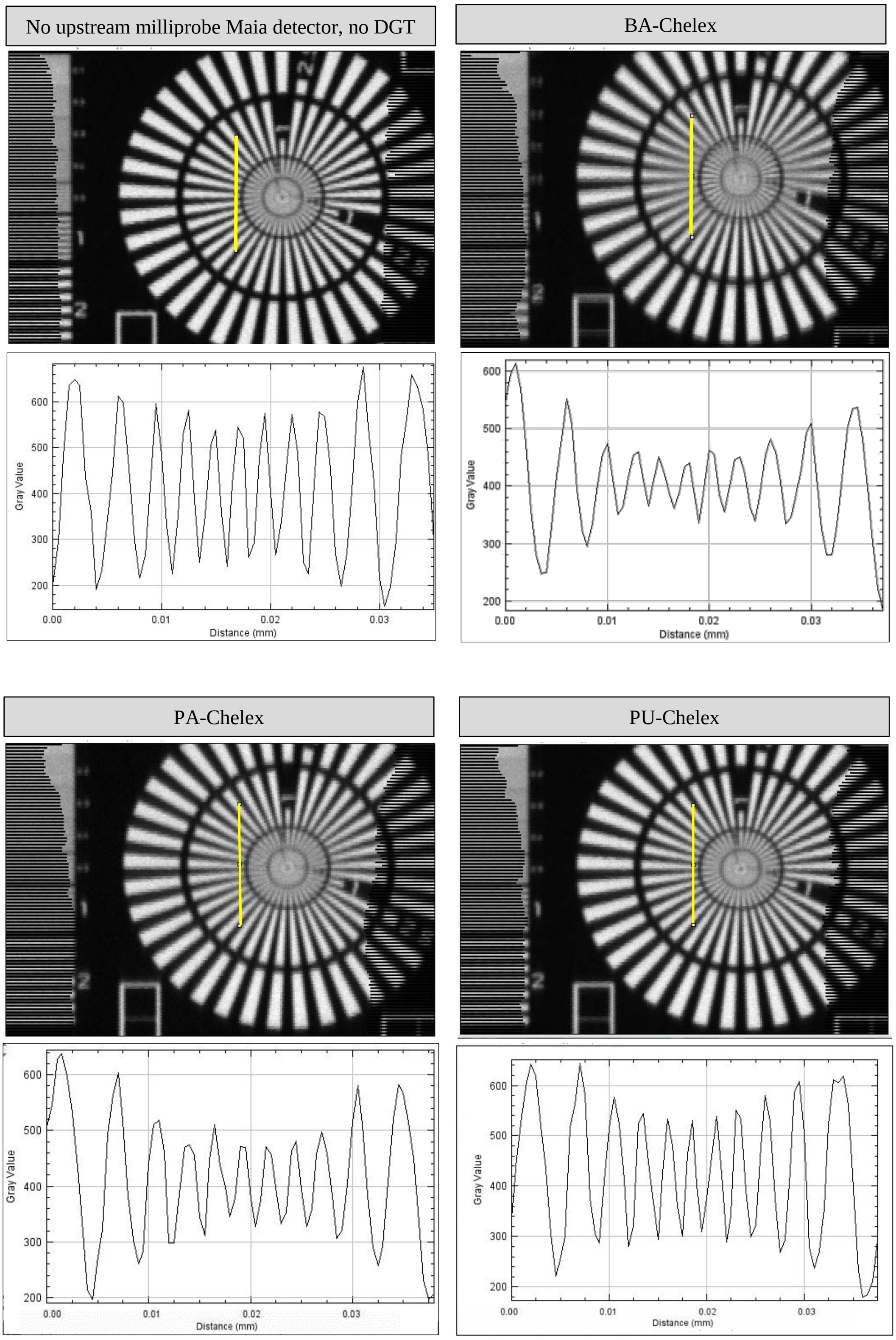

Figure S10: Vertical line profiles of the test pattern with no upstream sample and the upstream Maia milliprobe detector removed (top left), and equivalent line profiles while tandem scanning *bis*-acrylamide-Chelex, polyacrylamide-Chelex and polyurethane-Chelex gels upstream.

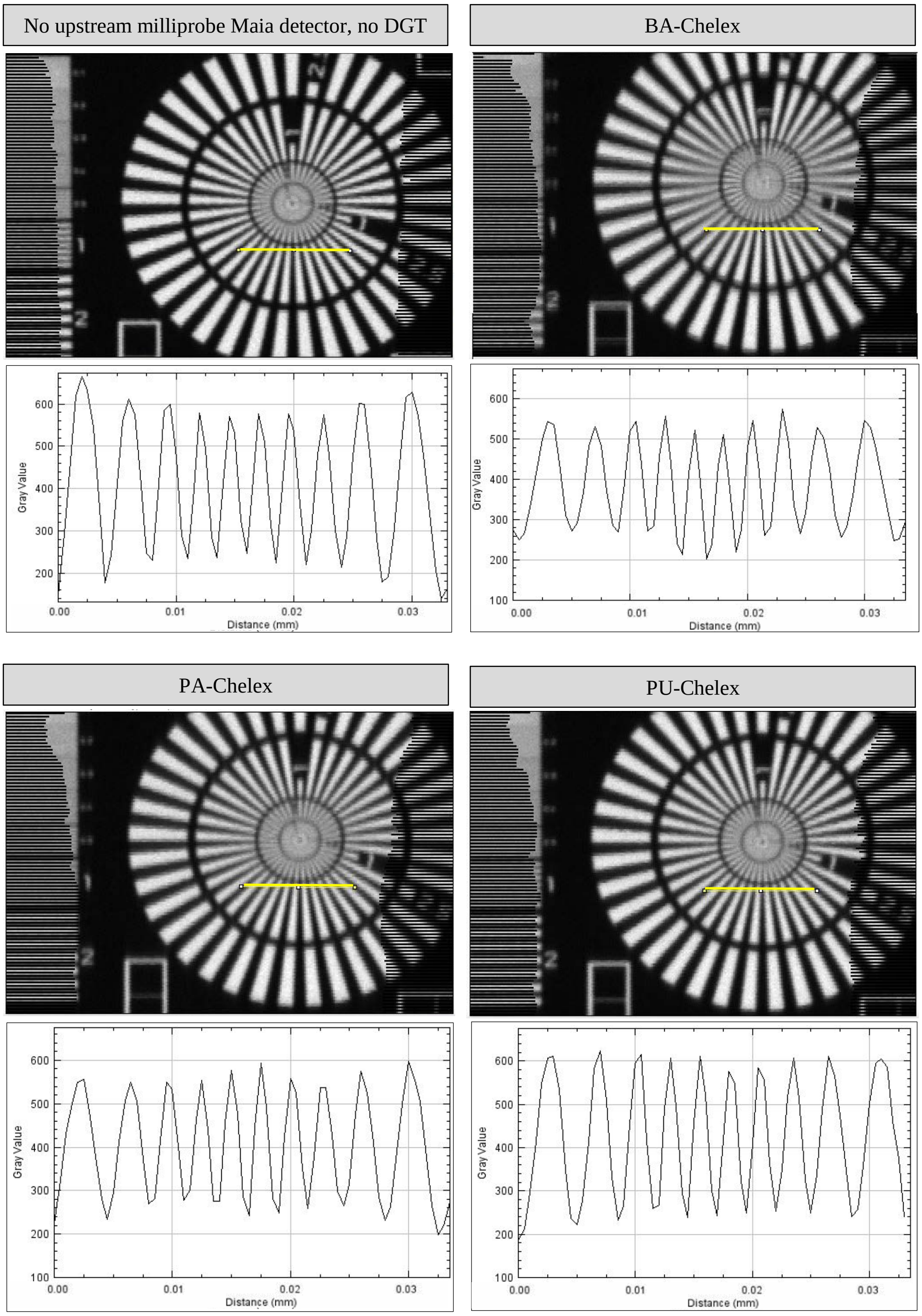

Figure S11: Horizontal line profiles of test pattern with no upstream sample and the upstream Maia milliprobe detector removed (top left) and equivalent line profiles while tandem scanning *bis*-acrylamide-Chelex, polyacrylamide-Chelex and polyurethane-Chelex gels upstream.

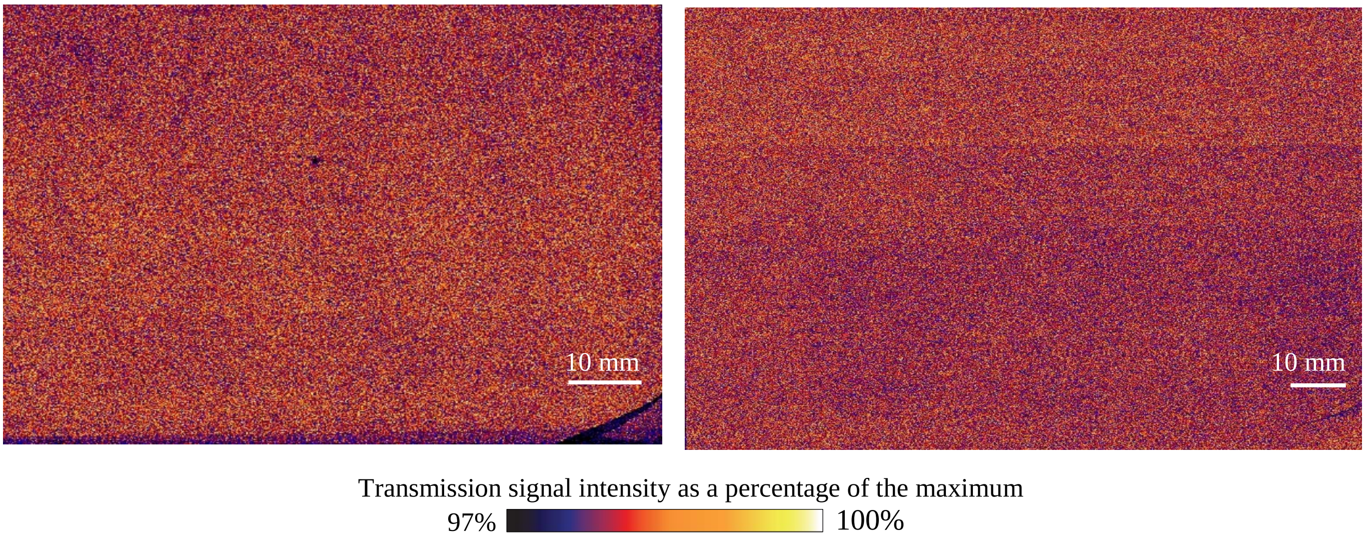

Figure S12. XFM images of polyurethane + Chelex–Metsorb (PU-CH-MS) (left) and polyurethane + Chelex (PU-CH) (right) gels showing the flux (transmission ion chamber signal). The minimum image value displayed (as a percentage of the maximum) was set at 97, while the maximum image value displayed was 100. Therefore, <3% variation in the flux during raster scanning can be observed. In the PU-CH-MS gel (left), the black area in the lower right corner of the image is due to crinkling/overlapping of the gel upon drying, producing a variation in transmittance >3%.

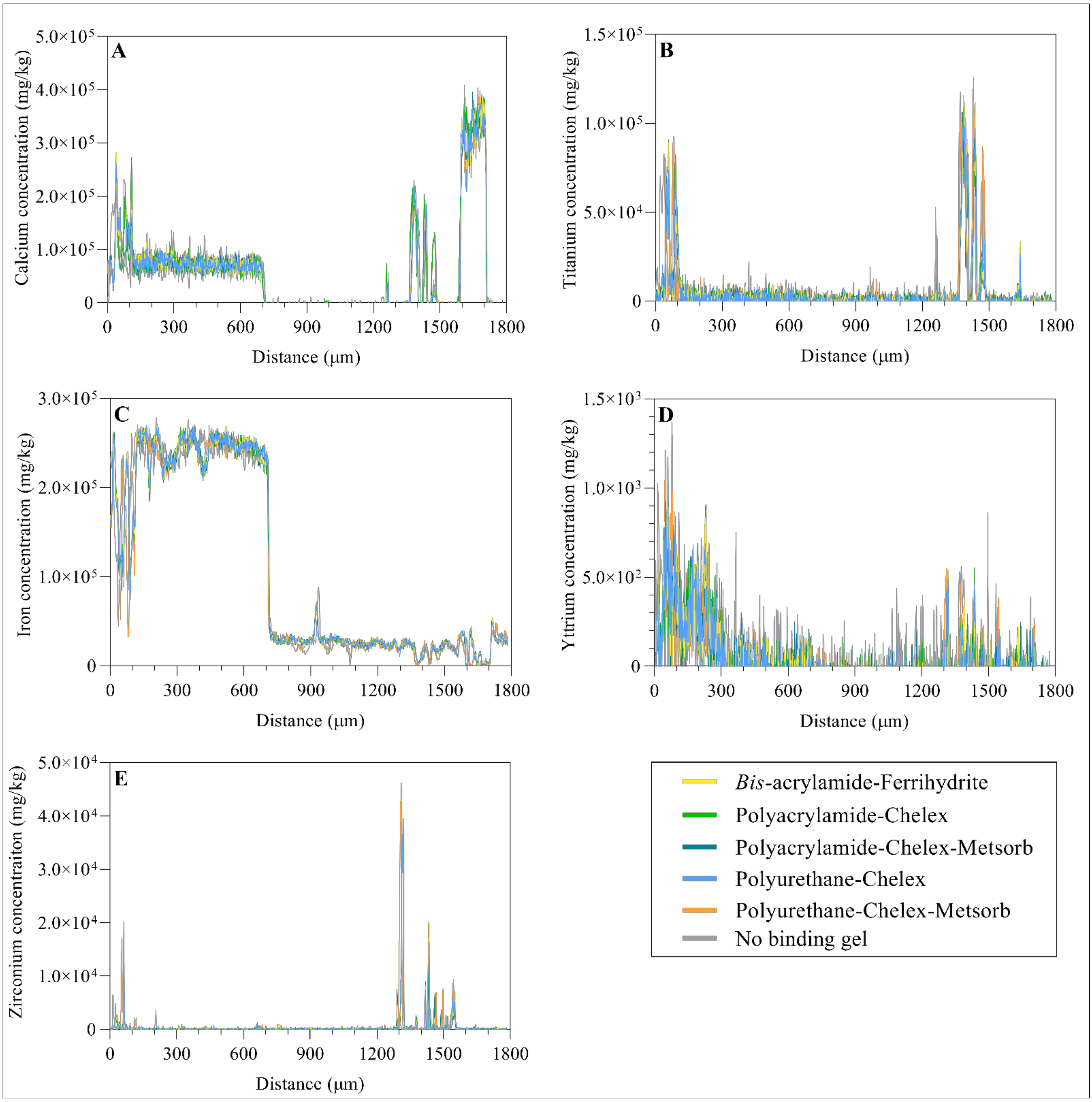

Figure S13. Concentrations (mg/kg) of calcium (a), titanium (b), iron (c), yttrium (d) and zirconium (e) in the area of the mineral sample indicated by the white rectangle in Figure 4c which was mapped at a pixel size of 1.0 µm.

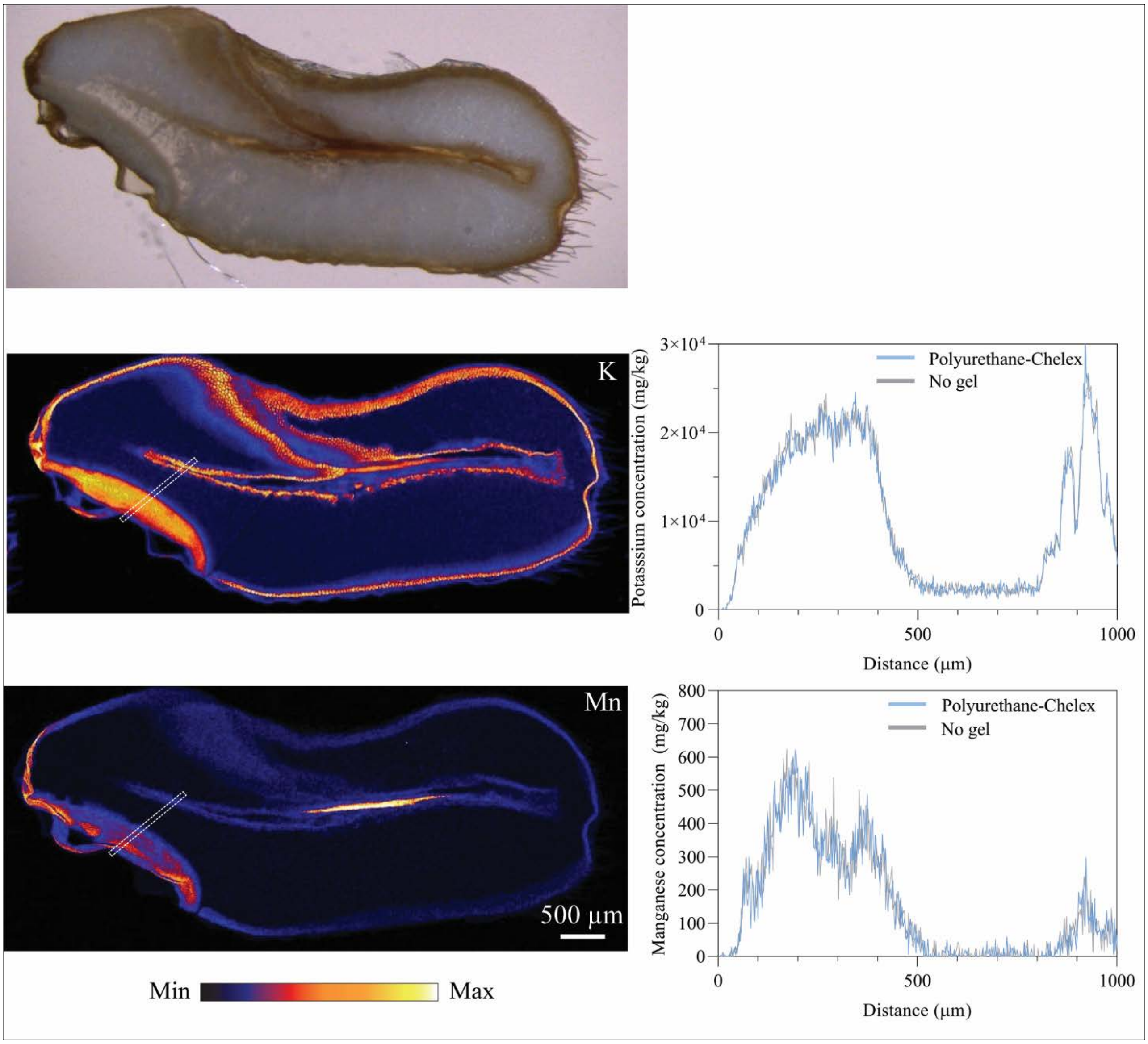

Figure S14. Optical scan of wheat grain longitudinal thin-section (top); elemental distribution of potassium (left middle) and manganese (left bottom); and, corresponding elemental concentrations (right) extracted from the dashed rectangular areas in the XFM images mapped with a pixel size of 2 µm.
